# Supplementary material for: Unravelling the structural complexity of glycolipids with cryogenic infrared spectroscopy
Source: Nat Commun. 2021 Feb 22;12:1201. doi: 10.1038/s41467-021-21480-1 (PMC7900115; doi:10.1038/s41467-021-21480-1)
Supplement: Supplementary file 1 — Supplementary Information [file 41467_2021_21480_MOESM1_ESM.pdf]

Unravelling the Structural Complexity of Glycolipids with Cryogenic Infrared Spectroscopy

Supplementary Information

Carla Kirschbaum[1,2], Kim Greis[1,2], Eike Mucha[2], Lisa Kain[3], Shenglou Deng[4], Andreas Zappe[1], Sandy Gewinner[2], Wieland Schöllkopf[2], Gert von Helden[2], Gerard Meijer[2], Paul B. Savage[4], Mateusz Marianski[5,6], Luc Teyton[3], Kevin Pagel[1,2]*

1 Institut für Chemie und Biochemie, Freie Universität Berlin, 14195 Berlin, Germany

2 Fritz-Haber-Institut der Max-Planck-Gesellschaft, 14195 Berlin, Germany

3 Department of Immunology and Microbiology, Scripps Research, La Jolla, CA 92037, USA

4 Department of Chemistry and Biochemistry, Brigham Young University, Provo, UT 84602, USA

5 Department of Chemistry and Biochemistry, Hunter College, The City University of New York, New York, NY 10065, USA

6 The PhD Program in Chemistry, Graduate Center, The City University of New York, New York, NY 10016, USA

Correspondence to: kevin.pagel@fu-berlin.de

Table of Contents

Abbreviations 2
List of Investigated Glycolipids 3
Ion Mobility-Mass Spectrometry and Tandem Mass Spectrometry 5
Supplementary IR Spectra 7
Synthetic Mixtures of α-and β-GalCer 10
Synthetic Mixtures of Monoglycosyl Phytosphingosines (4 Components) 13
Biological Application: Glycosylceramides 17
Computed IR Spectra and Structures 25

Abbreviations

Cer = Ceramide; Gal = Galactose; Glc = Glucose; GlcA = Glucuronic Acid; Gb3 = Globotriose; iGb3 = iso-Globotriose; DAG = Diacylglycerol

List of Investigated Glycolipids

Supplementary Table 1 Names, structures and exact masses of the investigated glycolipids.

**Glycosylceramides; exact mass: 809.7 u**	
α-GlcCer (d18:1/24:1(15*Z*))	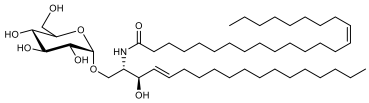
β-GlcCer (d18:1/24:1(15*Z*))	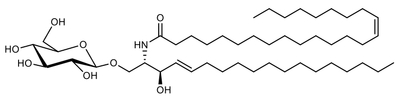
α-GalCer (d18:1/24:1(15*Z*))	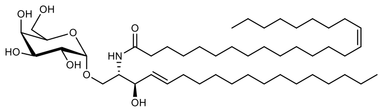
β-GalCer (d18:1/24:1(15*Z*))	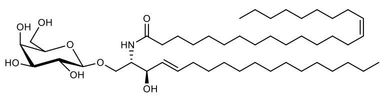
**Glycosylsphingosines; exact mass: 461.3 u**	
α-Glc sphingosine	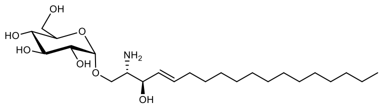
β-Glc sphingosine	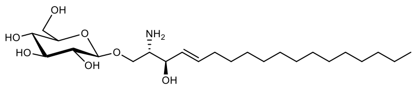
α-Gal sphingosine	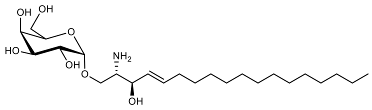
β-Gal sphingosine	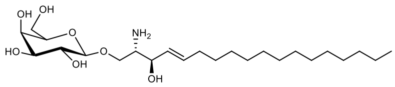
**Glycosylphytosphingosines; exact mass: 479.4 u**	
α-Glc phytosphingosine	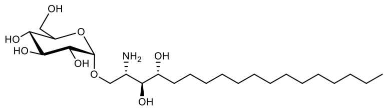
β-Glc phytosphingosine	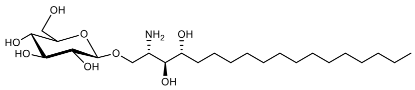
α-Gal phytosphingosine	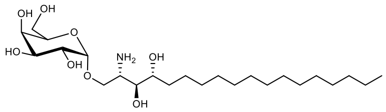
β-Gal phytosphingosine	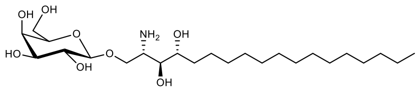

Glycosylphytosphingosines; exact mass: 493.3 u	
α-GlcA phytosphingosine	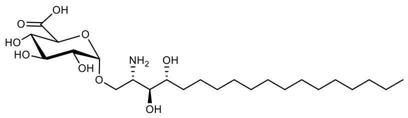
β-GlcA phytosphingosine	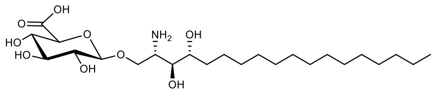

(iso-)Globotriaosylsphingosines; exact mass: 785.4 u	
β-Gb3 sphingosine	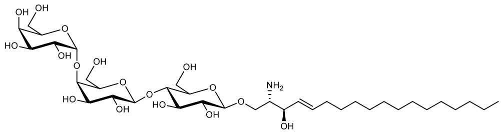
β-iGb3 sphingosine	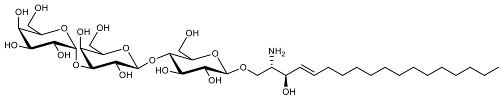

Globotriaosylceramide; exact mass: 1163.8 u	
α-Gb3Cer (d18:1/26:0)	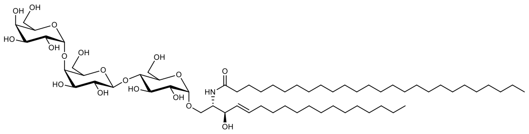

Galactosyl diacylglycerols; exact mass: 674.5 u	
α-Gal DAG (14:0/14:0)	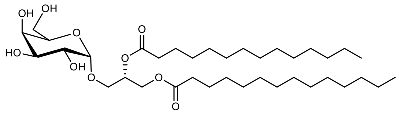
β-Gal DAG (14:0/14:0)	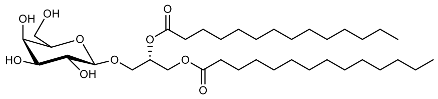

Ion Mobility-Mass Spectrometry and Tandem Mass Spectrometry

Supplementary Table 2 CCS of glycolipid adducts obtained from DT-IM-MS measurements (Helium, 2.2 Torr). The values are given in Å^2 (± 1 %). Only sodium adducts of β-Gb3- and β-iGb3 sphingosine and silver adducts of glycosylphytosphingosines exhibit distinguishable CCS. Silver adducts of glycosylphytosphingosines allow for the distinction of α- and β-glycosidic bonds.

Sample	$^{DT}CCS_{He}$ [M+H]$^+$	$^{DT}CCS_{He}$ [M+Na]$^+$	$^{DT}CCS_{He}$ [M+Ag]$^+$
α-GlcCer (d18:1/24:1(15*Z*))	234	233	233
β-GlcCer (d18:1/24:1(15*Z*))	235	233	233
α-GalCer (d18:1/24:1(15*Z*))	235	235	232
β-GalCer (d18:1/24:1(15*Z*))	234	235	233
α-Glc sphingosine	153	158	150
β-Glc sphingosine	152	157	153
α-Gal sphingosine	154	157	151
β-Gal sphingosine	153	158	150
α-Glc phytosphingosine	160	163	152
β-Glc phytosphingosine	161	164	157
α-Gal phytosphingosine	160	162	153
β-Gal phytosphingosine	161	164	156
α-GlcA phytosphingosine	160	163	152
β-GlcA phytosphingosine	161	163	156
β-Gb3 sphingosine	197	203	198
β-iGb3 sphingosine	199	198	196
α-Gb3Cer (d18:1/26:0)	281	283	280
α-Gal DAG (14:0/14:0)	–	207	202
β-Gal DAG (14:0/14:0)	–	210	204

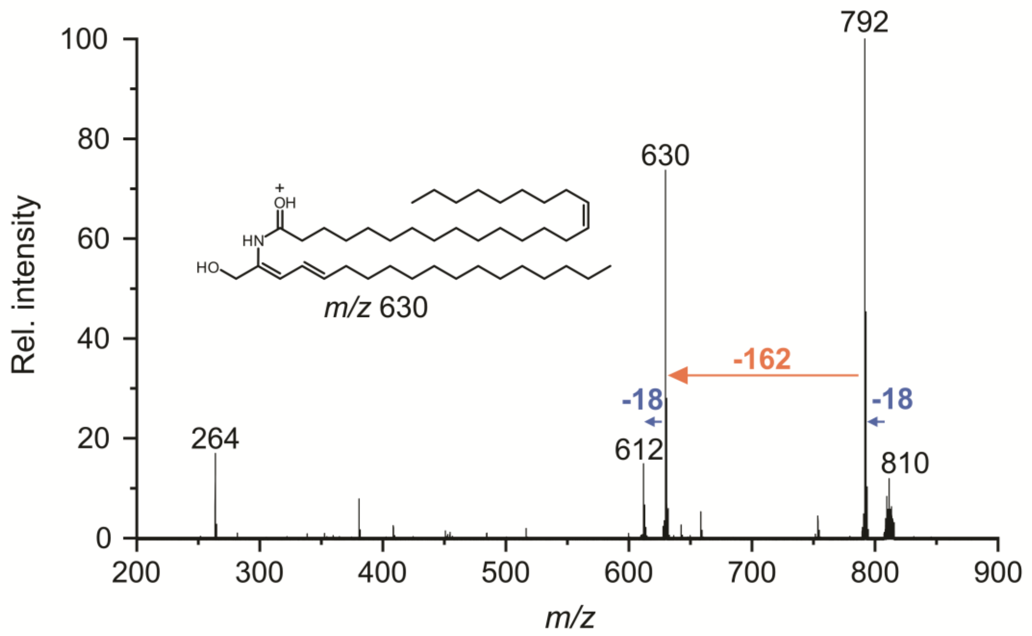

Supplementary Fig. 1 Exemplary MS/MS spectrum of β-GalCer $[M+H]^+$ (*m*/*z* 810) obtained by CID (20 V acceleration voltage). The main fragments result from water elimination (−18) and cleavage of the glycosidic bond (−162). The most abundant fragment is dehydrated ceramide (*m*/*z* 630, possible structure shown). The fragments yield no stereochemical information about the glycan and the MS/MS spectra of α- and β-GalCer are identical except for slightly varying relative intensities. Source data are provided as a Source Data file.

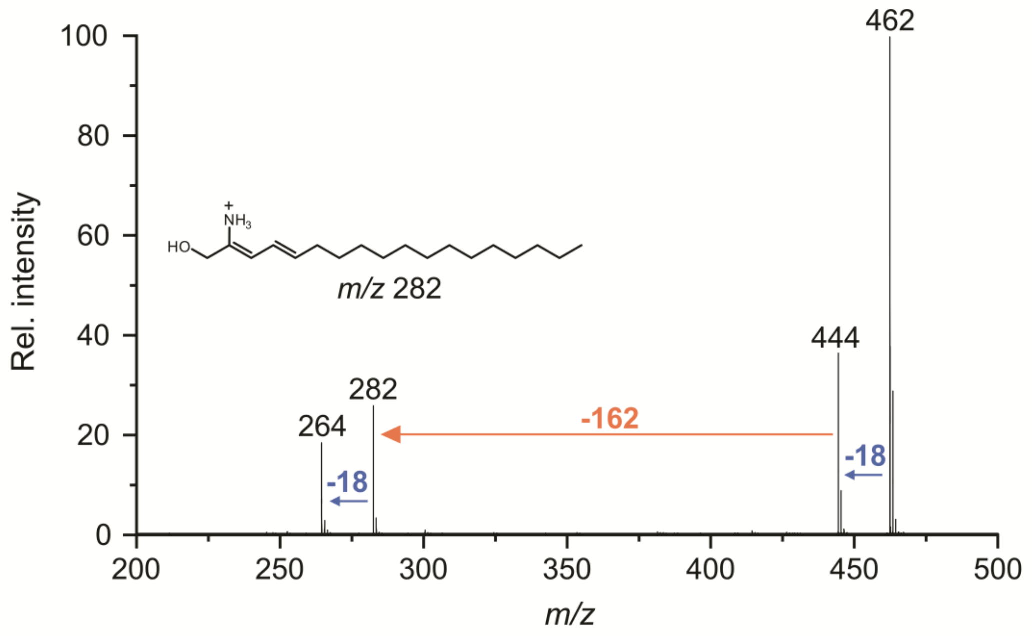

Supplementary Fig. 2 Exemplary MS/MS spectrum of α-Glc sphingosine $[M+H]^+$ (*m*/*z* 462) obtained by CID (10 V acceleration voltage). The main fragments result from water elimination and cleavage of the glycosidic bond yielding dehydrated sphingosine (*m*/*z* 282, possible structure shown). The fragments yield no stereochemical information about the glycan and the MS/MS spectra of all investigated glycosylsphingosines are indistinguishable. Source data are provided as a Source Data file.

Supplementary IR Spectra

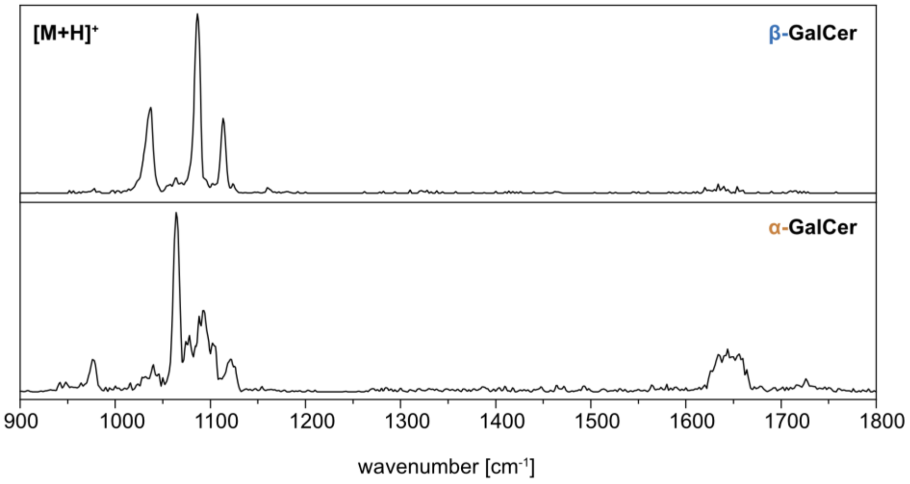

Supplementary Fig. 3 IR spectra of $[M+H]^+$ ions of α- and β-GalCer (d18:1/24:1(15*Z*)). The amide vibrations are partly suppressed compared to the spectra of the sodium adducts. Source data are provided as a Source Data file.

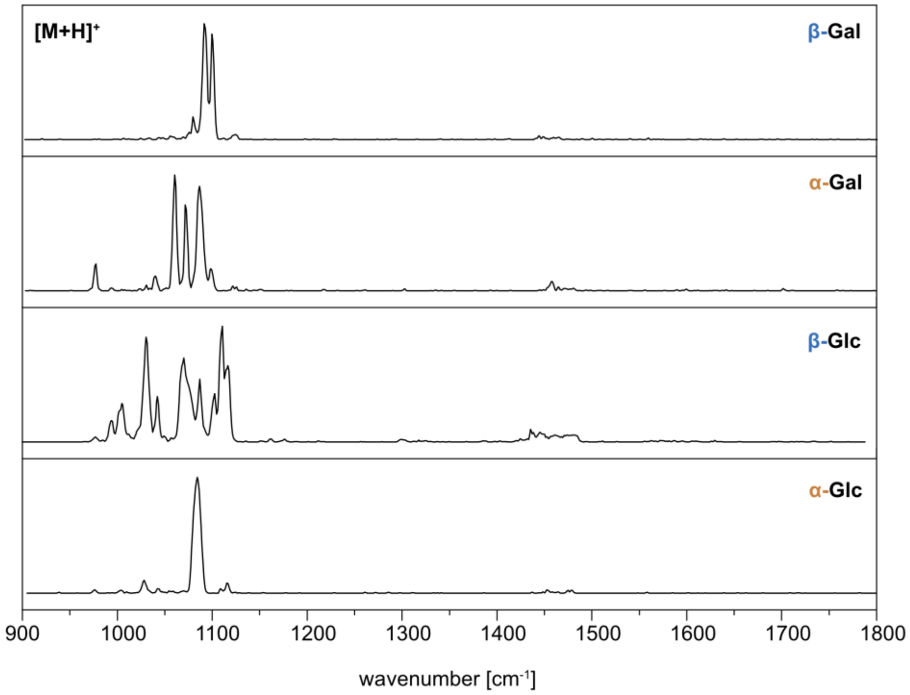

Supplementary Fig. 4 IR spectra of $[M+H]^+$ ions of glycosylsphingosines bearing Glc or Gal headgroups. The fingerprint regions are highly diagnostic and well-resolved. Weak NH_3^+ umbrella vibrations occur between 1400 and 1500 cm^{-1}. Source data are provided as a Source Data file.

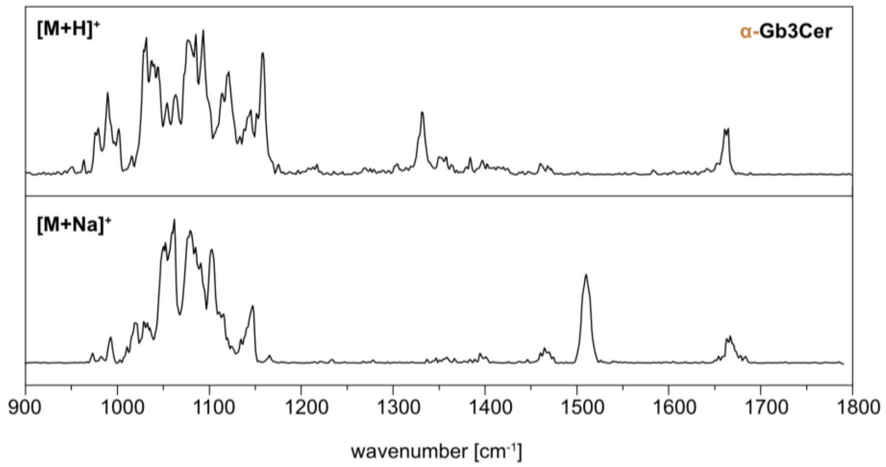

Supplementary Fig. 5 IR spectra of [M+H]+ and [M+Na]+ ions of α-Gb3Cer (d18:1/26:0). Distinct absorption bands in the fingerprint region are resolved despite the comparably large molecule size. Amide vibrations appear with varying intensity, depending on the cation. Source data are provided as a Source Data file.

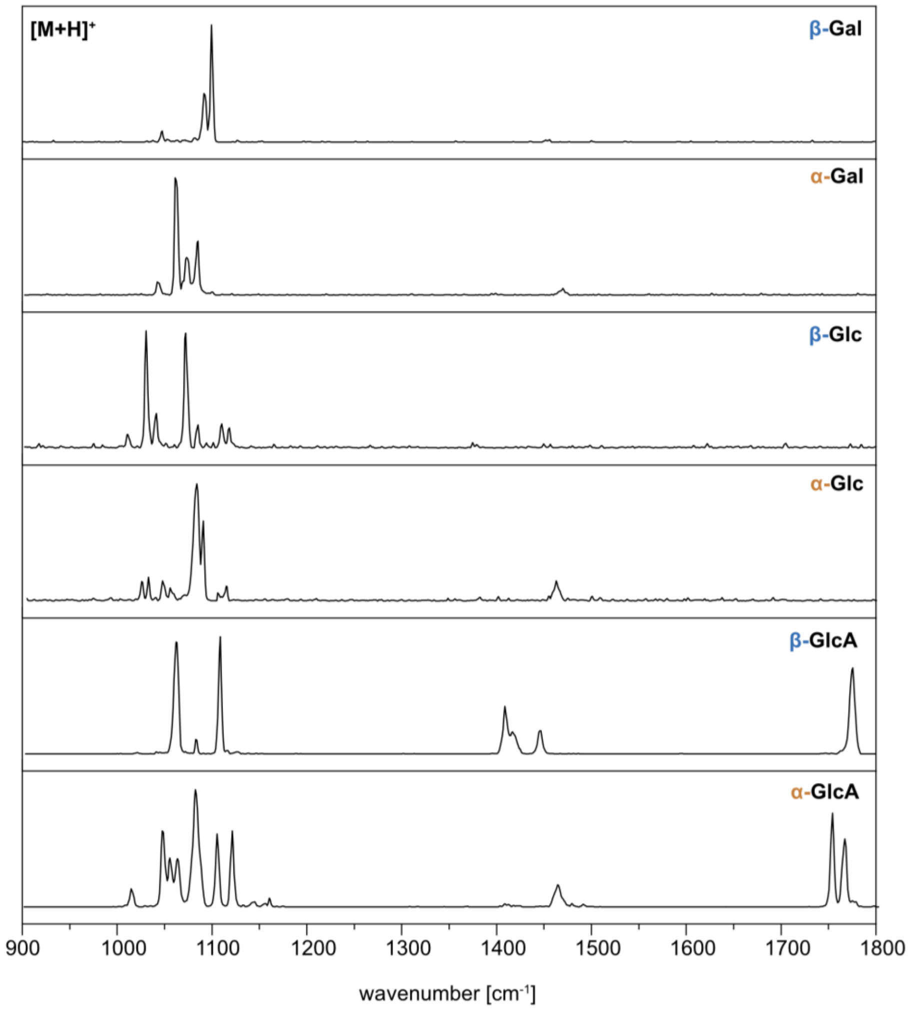

Supplementary Fig. 6 IR spectra of protonated glycosylphytosphingosines bearing Glc, Gal or GlcA headgroups. The fingerprint regions are highly diagnostic and well-resolved. Weak NH_3^+ umbrella vibrations occur between 1400 and 1500 cm^{-1}. The carboxyl groups of the GlcA headgroups yield characteristic C=O stretching vibrations above 1700 cm^{-1}. Source data are provided as a Source Data file.

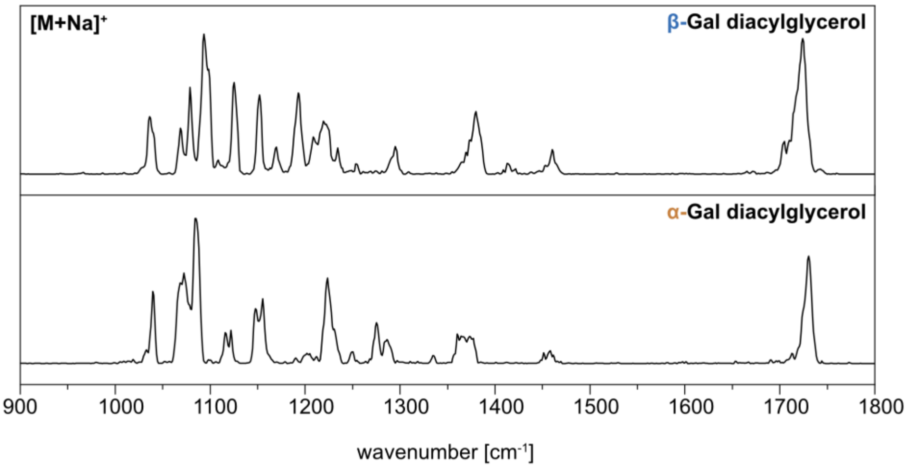

Supplementary Fig. 7 IR spectra of [M+Na]$^+$ ions of α- and β-galactosylglycerolipids (14:0/14:0). Compared to glycosphingolipids, more absorption bands appear above the fingerprint region between 1200 and 1500 cm^{-1}, and the ester groups yield characteristic C=O stretching vibrations beyond 1700 cm^{-1}. Source data are provided as a Source Data file.

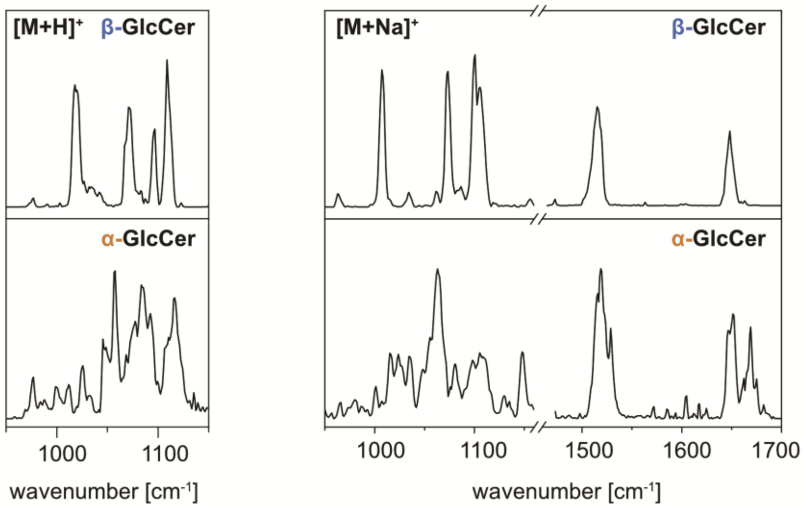

Supplementary Fig. 8 IR spectra of [M+H]$^+$ and [M+Na]$^+$ ions of α- and β-GlcCer (d18:1/24:1(15*Z*)) in the fingerprint region and the amide region. The fingerprint region of the sodiated species was employed for deconvolution of biological lipid extracts (*cf.* Supplementary Fig. 20). Source data are provided as a Source Data file.

Synthetic Mixtures of α-and β-GalCer

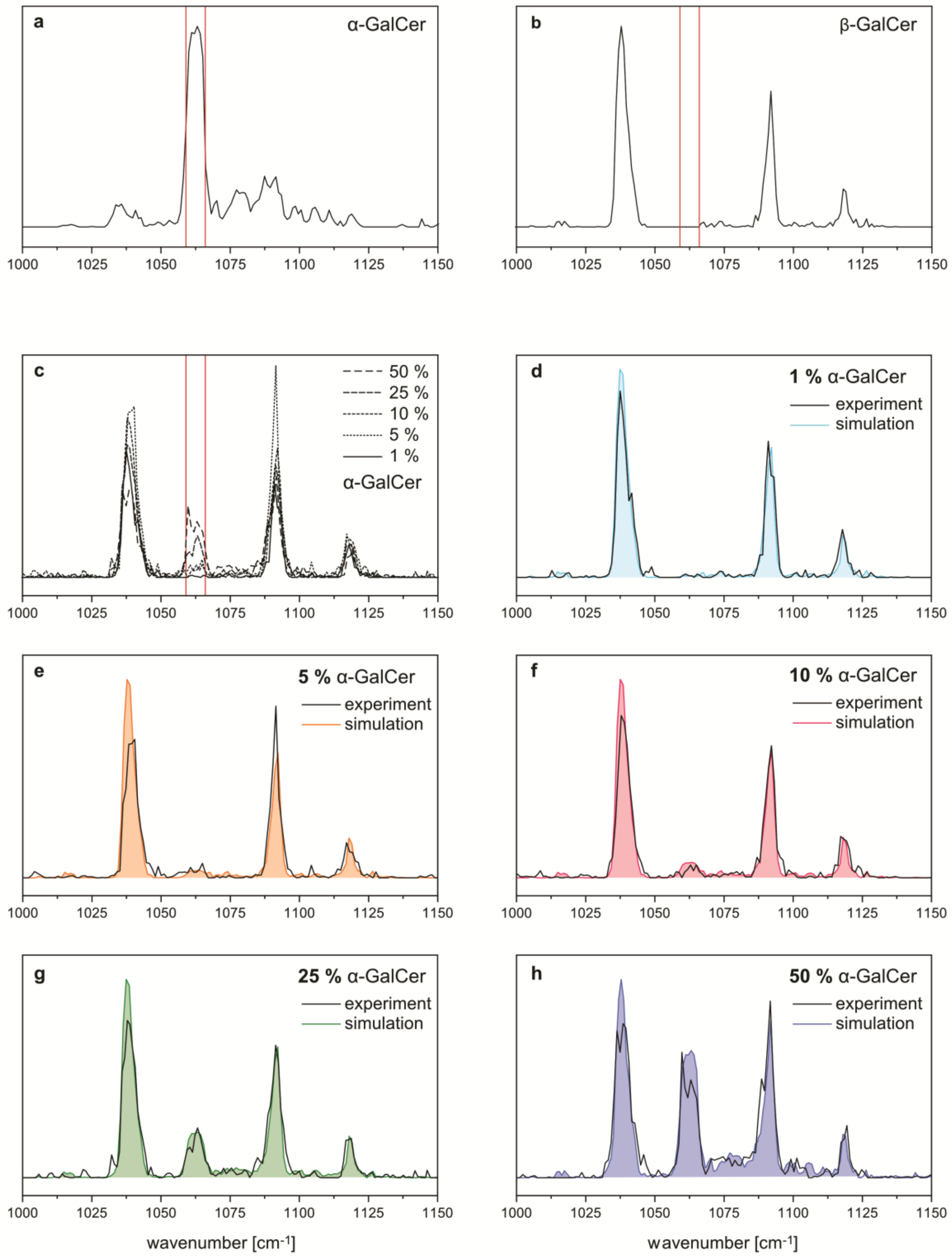

Supplementary Fig. 9 IR spectra of protonated α- and β-GalCer in the fingerprint region (1000–1150 cm^{-1}). The main absorption bands of the pure isomers do not overlap (**a**, **b**). The decrease of α-GalCer from 50 % to 5 % is accompanied by a roughly linear decrease of intensity of the absorption band within the set boundaries (1059–1066 cm^{-1}). At 1 %, α-GalCer cannot be detected anymore (**c**). The simulated mixture spectra generated by mathematically averaging the spectra of the pure isomers match the experimental spectra reasonably well (**d**–**h**). All spectra except for (**c**) are normalized. Source data are provided as a Source Data file.

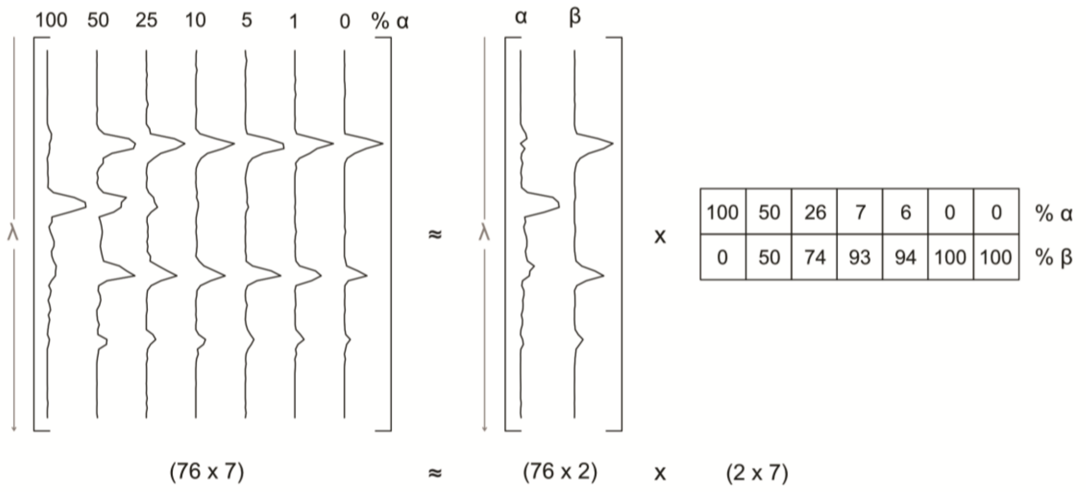

Supplementary Fig. 10*Graphical representation of non-negative matrix factorization (NMF) to deconvolute IR spectra of α- and β-GalCer. The (76 x 7) input matrix contains the binned experimental spectra from 1000–1150 cm^{-1}. The expected ratio of α-GalCer is indicated on top. The output contains the two spectra of pure α- and β-GalCer in a (76 x 2) matrix multiplied by their relative contributions to each of the input spectra.

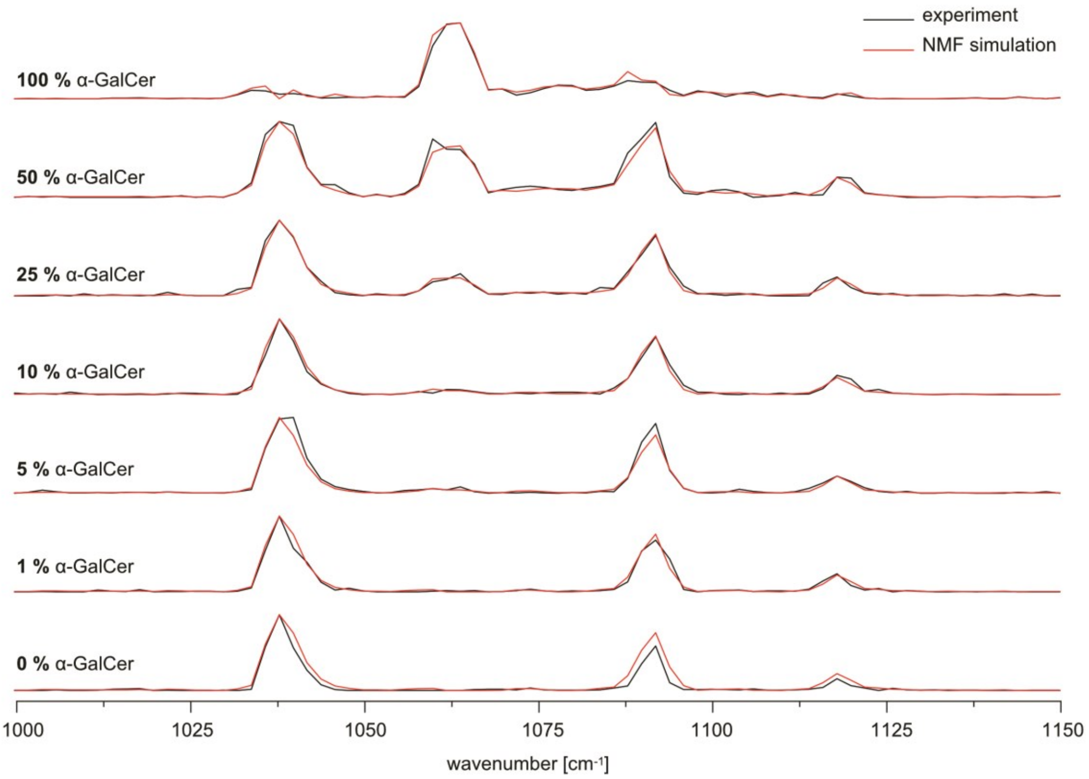

Supplementary Fig. 11: Comparison of NMF-simulated spectra of α- and β-GalCer (red) with the experimental IR spectra (black, input spectra from Supplementary Table 3). The spectra were simulated by matrix factorization of the output matrix (Supplementary Table 3) and the weighting factors depicted in Supplementary Fig. 10. Source data are provided as a Source Data file.

* The graphical representation of the input- and output IR spectra is based on the data in Supplementary Table 3.

Supplementary Table 3 Input- and output matrices of NMF containing normalized spectral intensities. Source data are provided as a Source Data file.

wavenumber	100 % α	50 % α	25 % α	10 % α	5 % α	1 % α	0 % α	α-GalCer	β-GalCer
	Input Matrix (76 x 7)							Output Matrix (76 x 2)	
1000	0	0	0	1.987	0	0	0	0.324	0
1002	0	1.071	0	0.656	0.890	0	0	0.420	0.177
1004	0	0.203	0	0.842	3.895	0.213	0	0.944	0
1006	0	1.090	1.096	0.419	1.933	0	0.545	0.872	0.088
1008	0	0	0	3.201	0.313	0	0	0.581	0
1010	0	0	2.677	0.898	0.186	0	0	0.642	0
1012	0	0	0.262	0	0.571	2.083	0.265	0.520	0
1014	0.514	0	1.230	0.383	0.183	0.433	1.278	0.528	0.095
1016	0.812	0	0.450	0	0.976	0.188	1.780	0.491	0.166
1018	1.087	0	0.115	0.286	0.889	2.447	2.152	0.911	0.034
1020	0.191	0	0.625	0	0.646	0	0	0.214	0.055
1022	0	0.530	4.712	0.435	1.572	0.064	0.112	1.279	0.077
1024	0.111	1.802	0.826	1.716	0.765	0.208	0	0.822	0.374
1026	0.101	0	0.073	1.495	0.265	0.515	0	0.388	0
1028	0	0.832	0.149	0.437	0.143	0.704	0	0.352	0.126
1030	0.635	0	0.087	0	0.151	0	0.137	0	0.287
1032	5.054	5.301	8.701	0.218	1.617	0.511	0	1.740	4.049
1034	10.855	18.875	10.767	11.162	4.700	4.937	1.579	6.390	9.244
1036	10.206	83.079	72.858	52.351	59.533	54.577	57.492	60.931	11.204
1038	5.796	100	100	100	98.039	100	100	100	0
1040	6.540	94.757	77.430	70.504	100	57.633	56.184	75.741	7.725
1042	4.548	39.544	37.784	30.224	46.085	38.663	26.642	36.566	2.148
1044	0.472	16.827	19.402	13.651	18.511	12.363	7.478	14.840	1.016
1046	1.150	16.455	4.209	6.106	8.387	2.102	1.197	5.718	3.846
1048	1.928	5.879	1.490	1.325	4.584	4.476	0	2.629	1.738
1050	1.330	0.755	0.103	0	2.175	0.955	0	0.547	0.604
1052	2.467	3.847	2.607	0.538	0.091	0	0	0.591	2.347
1054	2.368	0.580	1.087	0	0.802	0	0	0.072	1.316
1056	1.930	5.111	0.744	1.131	3.220	0	0	1.267	2.072
1058	15.936	17.054	6.508	3.603	4.122	0	0	2.089	12.400
1060	69.815	76.955	18.713	1.855	5.636	0.616	0	2.669	56.746
1062	98.325	63.576	21.762	6.569	4.113	1.415	0	0	66.343
1064	100	63.072	29.173	6.280	8.259	0.483	0	0.002	67.799
1066	58.237	44.159	12.878	3.904	2.011	1.514	0.933	0	41.466
1068	11.913	5.750	2.252	1.375	0	0	1.235	0	7.343
1070	12.403	10.030	1.752	0.971	0.625	0.065	1.282	0.078	8.900
1072	4.664	12.837	4.551	1.786	0.403	1.440	0.632	2.187	5.730
1074	7.971	14.461	3.412	1.027	0.960	3.437	2.896	2.413	7.452
1076	13.480	13.134	4.937	2.089	0.362	1.127	0.750	1.029	10.280
1078	17.892	10.274	2.055	2.980	0	0.186	0.086	0	11.454
1080	17.370	9.073	3.944	2.958	0.570	1.162	0.064	0	11.046
1082	10.952	11.731	2.249	2.137	0.177	0.799	0.362	0.647	8.621
1084	12.059	14.195	10.964	0.516	2.324	1.456	0.511	2.490	9.933
1086	16.448	18.077	9.797	8.531	1.920	2.884	3.046	4.129	12.328
1088	23.603	58.481	31.496	20.995	19.179	12.902	5.968	19.150	24.235
1090	21.561	77.617	54.664	49.164	67.319	53.761	35.103	53.025	16.492
1092	21.434	98.957	79.805	76.250	92.087	68.444	58.700	76.216	15.516
1094	11.064	26.901	38.166	39.116	30.186	43.335	10.295	30.839	3.576
1096	4.278	5.443	12.831	11.980	6.060	3.096	1.159	6.322	2.217
1098	10.114	3.781	3.188	2.946	0.717	0	0.884	0.306	5.905
1100	7.646	8.698	2.220	2.740	0.191	1.261	1.553	1.197	5.904
1102	2.804	10.328	0.672	0.141	0.962	1.708	0.960	1.476	4.005
1104	6.476	6.973	1.861	0	4.912	2.358	0.761	1.709	4.560
1106	8.643	0.014	2.507	0.865	2.138	0.184	1.928	0.143	4.001
1108	2.927	0.826	0.052	0.213	0.782	1.078	0.222	0.118	1.506
1110	6.250	2.214	0	1.955	0.247	0.978	0.146	0	3.489
1112	3.500	6.327	0	0.358	1.784	0.737	0.560	0.824	3.253
1114	1.918	2.654	0.285	0.517	6.001	3.287	1.400	2.262	0.725
1116	1.087	2.912	17.008	7.911	13.231	14.156	3.812	10.018	0
1118	6.004	26.343	24.842	25.245	22.444	23.817	15.364	22.303	3.675
1120	3.461	25.339	10.843	20.363	15.438	8.161	6.002	13.424	5.047
1122	0.502	5.620	3.855	4.230	6.894	2.093	3.079	4.281	0.560
1124	0	3.591	1.889	6.714	2.087	4.172	0	3.131	0
1126	0	1.628	3.054	1.963	0.600	0	2.577	1.579	0.120
1128	0	0	0.356	0.922	2.160	1.558	0.792	0.976	0
1130	0	0	0.891	0.302	0	0.633	0.299	0.344	0
1132	0	0	1.067	0	0	0	0.435	0.244	0
1134	0	0.509	1.103	0.298	0.695	0	0	0.438	0.088
1136	0.363	0.594	0	0.588	0.045	0.081	0.111	0.160	0.288
1138	0.892	0	0.819	0.185	0	0	0.061	0.052	0.452
1140	0	0.439	0.319	0	0	0.053	0	0.108	0.126
1142	0	0.298	2.500	0	0	0.144	0	0.470	0.129
1144	2.082	0.822	0	0	0.956	0	0	0	1.196
1146	0.763	0	2.360	0	0.320	0	0	0.349	0.413
1148	0	0.262	0	0	1.868	0	0	0.401	0
1150	1.088	1.887	0	0.199	0	0	0	0.075	1.068

Synthetic Mixtures of Monoglycosyl Phytosphingosines (4 Components)

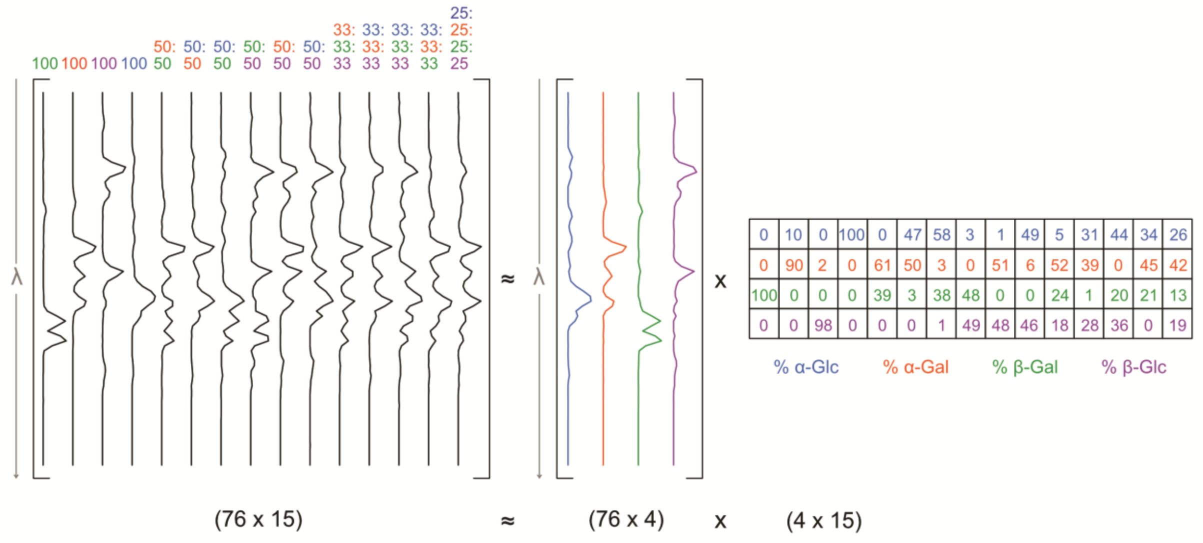

Supplementary Fig. 12*Graphical representation of NMF to deconvolute IR spectra of α- and β-Glc/Gal phytosphingosine. The (76 x 15) input matrix contains the experimental spectra. The expected ratio of the four isomers is indicated on top. The output contains the four spectra of pure α- and β-Glc/Gal phytosphingosine in a (76 x 4) matrix multiplied by their relative contributions to each of the input spectra.

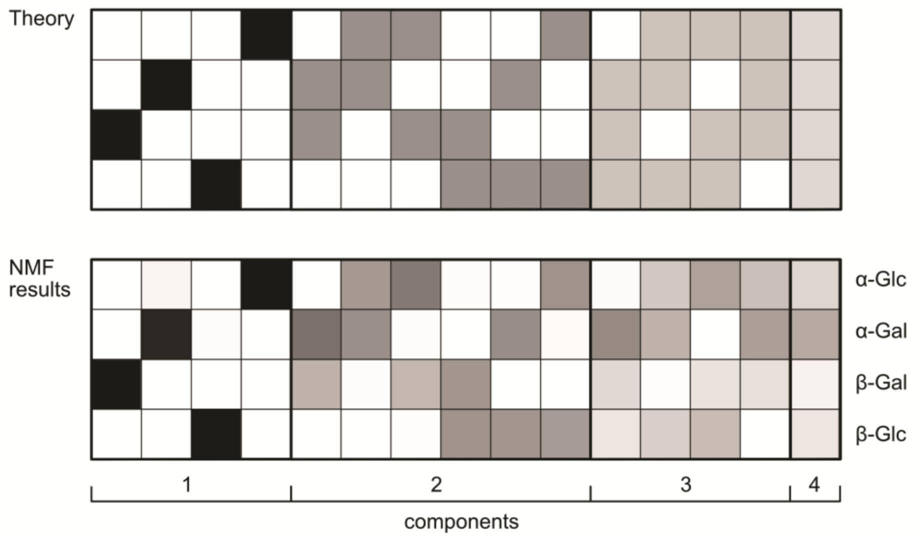

Supplementary Fig. 13 Heatmap representation of the weighting factors predicted by the mixing ratios (top) and calculated by NMF from the 15 experimental spectra (bottom). The weighting factors in the (4 x 15) matrix from Supplementary Fig. 12 were converted into the corresponding ratio of black and white (white = 0 %, black = 100 % glycolipid).

* The graphical representation of the input- and output IR spectra is based on the data in Supplementary Tables 4–5.

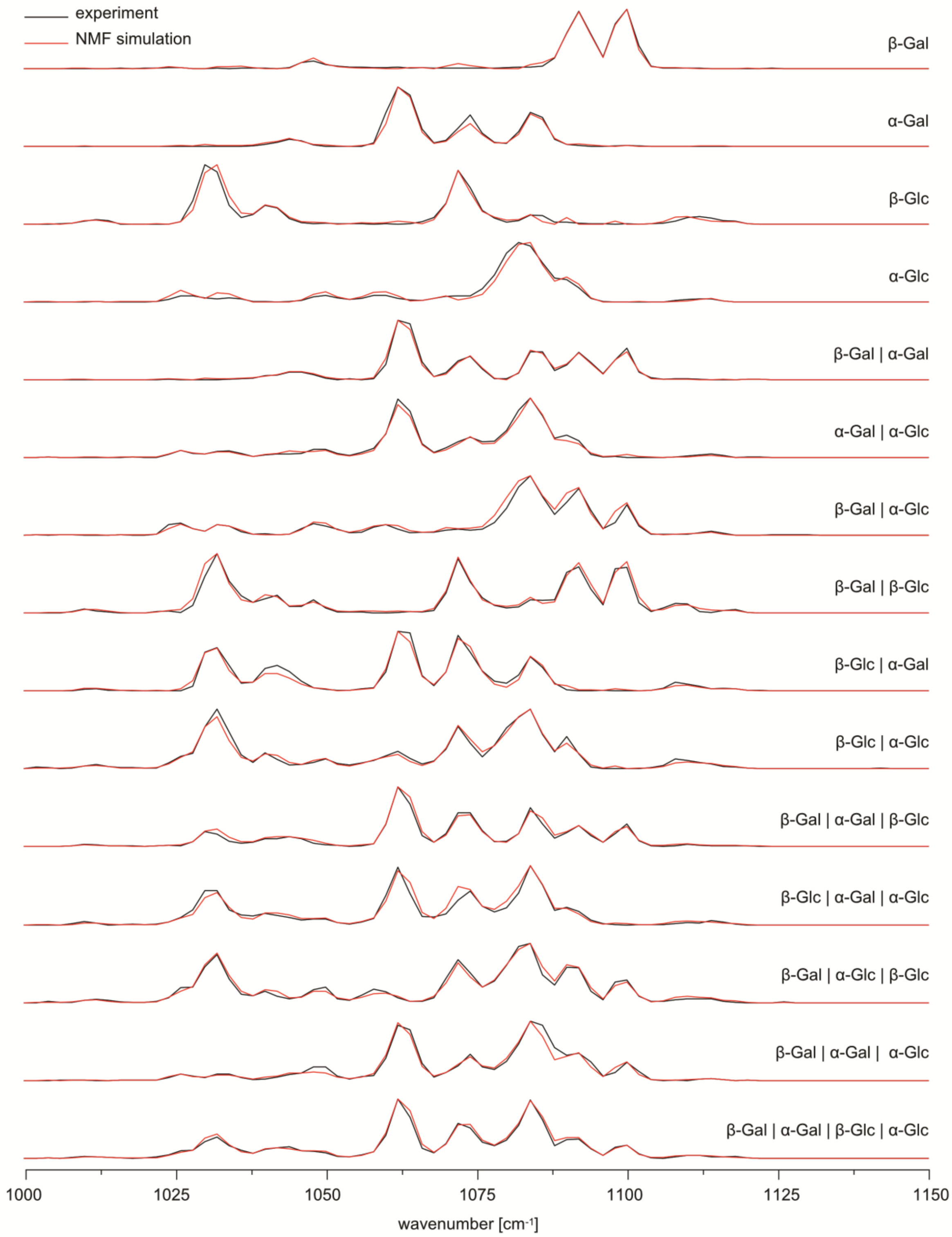

Supplementary Fig. 14 Comparison of NMF-simulated spectra of Glc/Gal phytosphingosines (red) with the experimental IR spectra (black, input spectra from Supplementary Table 4). The spectra were simulated by matrix factorization of the output matrix (Supplementary Table 5) and the weighting factors given in Supplementary Fig. 12. Source data are provided as a Source Data file.

Supplementary Table 4 Input matrix for NMF containing normalized spectral intensities. Source data are provided as a Source Data file.

wn	β-Gal	α-Gal	β-Glc	α-Glc	β-Gal α-Gal	α-Gal α-Glc	β-Gal α-Glc	β-Gal β-Glc	β-Glc α-Gal	β-Glc α-Glc	β-Gal α-Gal β-Glc	β-Glc α-Gal α-Glc	β-Gal α-Glc β-Glc	β-Gal α-Gal α-Glc	β-Gal α-Gal β-Glc α-Glc
	Input Matrix (76 x 15)														
1000	0	0	0	0.011	0	0	0.058	0	0	0	0	0.033	0	0	0
1002	0.023	0	0	0.138	0	0.325	0.711	0	0	2.234	0	0.139	0.257	0	0.132
1004	0.001	0	0.071	0.158	0.048	0.830	0.315	0.036	0	1.954	0	0.050	2.266	0	0.532
1006	0	0	0.638	0	0.007	0.237	0.590	0.255	0	0.064	0.004	0	1.065	0.897	0.328
1008	0.011	0	1.705	0.019	0	0	0.731	2.416	0	0.728	0.581	2.276	1.224	1.180	2.072
1010	0.019	0	5.106	0.337	0	0	0.257	6.477	2.505	3.990	3.261	5.690	4.301	0.527	3.772
1012	0.104	0.031	7.997	0.770	0.035	0	0	3.554	3.121	6.711	2.526	3.093	5.981	0	2.387
1014	0.320	0.016	5.912	0.091	0.151	0.054	0	2.029	1.211	3.531	0.873	0.307	4.304	0.187	1.713
1016	0.467	0	0.062	0.188	0.084	0.084	0.525	0.344	0.772	1.548	0.616	0.984	1.780	0.725	1.062
1018	0.305	0.023	0.030	0.389	0.172	0.655	0.245	0	0.251	1.399	0.749	1.305	0.490	0.960	0.142
1020	0.577	0.040	0.081	0.410	0.167	0.296	0.258	0	0	0.675	0.289	1.233	0.488	0.594	1.264
1022	0.934	0	0.042	0.561	0.077	0.160	1.280	2.187	0	0.599	0.504	2.044	2.312	0.442	2.366
1024	1.664	0.014	0.345	4.395	0.427	5.286	18.270	3.057	0.006	9.256	1.645	5.278	8.415	4.793	6.380
1026	1.562	0.002	4.133	10.102	0.074	12.240	20.400	1.268	0.141	20.621	0.942	12.588	25.180	11.218	12.512
1028	0.254	0.007	39.020	10.386	0.019	6.572	11.394	12.612	7.880	25.357	7.512	25.400	26.408	7.968	13.613
1030	0.422	0.038	100	6.804	0.022	6.257	6.671	61.018	64.681	69.750	25.112	57.890	60.274	5.496	28.559
1032	0.529	0.014	87.493	5.082	0.008	9.910	18.105	100	72.775	99.384	20.333	57.898	81.068	11.104	36.022
1034	0.114	0.041	31.817	6.453	1.227	11.247	15.268	53.542	42.669	61.104	8.827	25.406	40.139	11.157	21.153
1036	0.536	0.008	10.725	4.775	1.925	7.236	5.928	29.225	12.496	23.028	4.911	16.716	14.536	4.710	13.243
1038	1.004	0.438	16.530	0.516	2.275	1.913	1.797	17.795	14.492	10.104	6.563	14.987	11.553	1.825	8.729
1040	0.762	3.433	31.263	0.774	7.134	4.516	2.704	23.806	37.295	26.572	12.669	19.950	19.831	2.868	15.445
1042	0.375	7.164	27.667	0.280	7.274	6.327	1.206	29.846	42.390	15.418	12.588	16.327	11.442	5.784	17.639
1044	1.832	12.018	9.109	0.727	12.838	6.036	2.061	11.001	32.500	6.360	16.149	12.453	6.417	10.064	19.695
1046	10.581	9.284	2.272	2.262	12.973	7.214	11.271	10.796	16.086	6.569	12.915	8.717	10.301	13.083	12.696
1048	12.969	2.180	1.030	8.598	8.265	13.710	19.338	22.580	5.194	9.982	5.469	9.951	22.396	22.793	12.190
1050	6.543	0.266	1.936	10.698	2.606	13.373	15.405	8.822	2.478	15.887	2.774	10.520	26.403	23.122	9.705
1052	4.037	0.097	0.713	6.785	0.293	7.021	9.923	2.017	0.544	8.585	1.398	3.948	6.994	7.798	3.182
1054	2.947	0.029	1.201	4.248	2.016	3.704	4.337	1.751	0.643	6.160	0.262	1.822	3.958	2.976	2.387
1056	2.246	0.146	0.724	6.052	0.300	8.108	4.981	1.399	3.817	9.023	0.892	5.170	12.515	6.458	6.248
1058	1.923	6.020	0.154	11.239	2.060	15.607	13.263	0.127	4.783	13.586	4.401	11.085	23.014	8.392	11.129
1060	1.822	56.294	1.221	11.038	25.614	39.606	17.708	0.039	31.787	21.517	36.871	49.215	19.177	37.698	38.352
1062	2.467	100	0.195	6.060	100	97.901	9.949	0.928	100	28.893	100	96.984	8.229	92.862	100
1064	1.223	85.771	0.449	3.391	93.717	77.744	4.656	2.763	97.445	16.722	70.957	53.012	2.890	85.186	68.901
1066	1.387	28.924	4.377	2.250	29.647	22.671	5.169	1.492	25.339	7.922	18.250	14.919	2.707	28.985	17.155
1068	1.072	5.960	11.463	5.971	5.406	8.731	5.263	4.567	8.211	12.789	6.699	13.757	8.899	6.219	9.264
1070	1.220	9.904	34.693	9.781	12.150	15.879	8.543	32.970	31.177	32.487	20.959	19.146	41.040	13.365	25.015
1072	1.244	30.685	90.488	9.898	31.201	26.597	10.641	91.175	92.892	70.383	56.326	41.708	72.080	24.397	58.494
1074	1.085	52.536	61.620	9.813	39.067	34.149	8.116	52.062	65.198	42.233	56.425	57.108	50.361	44.359	51.201
1076	1.203	22.354	23.412	21.798	18.115	26.865	10.379	25.280	33.214	19.858	26.293	31.727	26.103	22.931	27.404
1078	0.841	6.611	10.213	45.562	1.523	27.687	21.109	12.470	16.531	39.430	7.937	21.783	36.335	18.691	21.702
1080	2.035	4.855	3.785	82.283	1.953	45.335	45.503	8.212	12.654	68.079	8.852	28.848	63.184	32.631	30.808
1082	2.840	24.843	6.109	100	11.287	79.181	80.897	10.590	26.761	86.548	20.969	53.320	94.432	61.030	55.380
1084	2.319	57.566	15.238	93.933	46.988	100	100	21.994	57.078	100	64.989	100	100	100	98.302
1086	4.077	48.754	14.165	65.884	46.630	69.900	64.560	21.471	39.238	57.193	37.726	67.423	46.867	93.244	64.682
1088	18.312	12.250	3.594	39.784	15.312	32.824	34.900	22.434	12.960	28.214	14.015	24.362	29.687	55.055	21.433
1090	59.558	3.532	3.220	37.456	24.913	37.597	55.849	68.053	1.346	53.548	22.940	29.751	59.756	42.968	31.369
1092	96.064	1.367	1.299	23.598	46.391	28.121	78.752	77.808	0.362	25.157	34.919	22.869	58.642	46.171	32.792
1094	57.877	0.223	0.867	6.392	28.960	5.130	41.671	39.909	0.104	2.276	17.776	8.383	19.263	31.303	16.045
1096	19.595	0.314	0.611	0.600	10.287	0.706	11.225	13.441	0.642	0.150	7.044	2.339	4.782	7.933	5.155
1098	75.477	0.494	0.387	0.348	33.638	1.492	21.131	74.054	0.332	0.074	21.355	0.453	34.699	13.801	19.094
1100	100	1.397	0.859	0.087	52.480	0.136	51.368	76.432	0.028	0.221	37.247	0.042	38.411	30.932	22.198
1102	38.772	0.498	0.073	0.055	12.394	0.142	15.730	17.079	0.051	0.141	9.479	0.545	11.311	15.272	7.904
1104	4.265	0	0.629	0.025	3.168	0.031	1.825	2.786	1.106	1.915	0.461	0.684	2.433	1.575	1.198
1106	0.607	0.009	1.964	0.293	0.608	0.166	2.023	8.356	4.382	5.080	0	0.687	2.654	0.318	0.351
1108	0.713	0.032	4.859	2.371	2.072	0.488	2.062	15.967	14.148	16.051	1.132	2.752	4.796	0.473	2.250
1110	0.704	0.006	10.864	4.113	1.717	2.205	1.910	15.123	11.120	14.055	3.497	4.451	6.684	0.241	5.183
1112	0.619	0	13.210	4.889	0.623	3.959	4.292	2.704	8.028	10.038	1.665	3.320	7.165	2.508	5.255
1114	0.052	0	10.119	5.444	0.145	5.882	7.061	1.270	3.380	7.345	1.217	7.389	6.442	3.330	2.710
1116	0.008	0	5.707	1.514	0.014	3.075	3.689	3.908	4.420	3.564	0.835	5.190	2.746	1.465	2.661
1118	0	0.005	4.903	0.246	0.209	0.085	0.319	5.562	3.499	2.832	0.516	1.562	0.947	0.331	3.244
1120	0.254	0.183	1.022	0	0.901	0.453	0.319	1.135	0.510	0.603	0.629	0.184	0.047	0.475	0.644
1122	0.350	0.351	0	0	0.917	0.788	0.192	0	0.698	0.114	0.030	0.033	0.010	0.380	0
1124	0.473	0.031	0	0	0.076	0.276	0.819	0.080	0.423	0	0.006	0	0.393	0.070	0.008
1126	0.171	0	0	0	0.006	0.027	0.524	0.087	0.229	0.191	0.082	0	1.397	0	0.072
1128	0	0	0	0.027	0.005	0.256	0.651	0.022	0	0.072	0.018	0.059	0.104	0.134	0.035
1130	0	0.008	0	0.135	0	0.152	0.720	0.094	0.067	0	0	0.146	0	0.123	0.045
1132	0	0.218	0	0	0	0	0.064	0.011	0.115	0	0	0.015	0	0	0.141
1134	0	0.364	0	0	0.018	0	0	0	0.008	0	0	0	0	0	0.076
1136	0.008	0.058	0	0	0.031	0	0	0	0	0.384	0	0.055	0.042	0.043	0.035
1138	0.033	0	0	0	0	0.119	0	0	0.068	0.115	0	0.017	0.039	0.193	0.105
1140	0.058	0	0	0	0	0.095	0	0	0.295	0.309	0.012	0.011	0	0.083	0.033
1142	0.006	0	0	0.295	0	0	0	0	0.068	0.446	0.056	0.124	0.019	0.041	0
1144	0	0	0	0.305	0.061	0.021	0	0.064	0.018	0	0	0.064	0.082	0.022	0
1146	0	0	0	0.018	0.078	0.022	0	0.215	0.275	0	0	0.062	0.077	0	0
1148	0	0	0	0.010	0.080	0.028	0	0.122	0.052	0	0	0.400	0.073	0	0.162
1150	0	0	0.131	0.254	0.007	0.018	0	0.061	0.391	0	0	0.149	0.257	0	0.111

Supplementary Table 5 Output matrix of NMF containing the spectra of the pure isomers.

wavenumber	α-Glc	α-Gal	β-Gal	β-Glc
	Output Matrix (76 x 4)			
1000	0	0.015	0	0
1002	0	0.504	0	0.198
1004	0	0.725	0	0.374
1006	0	0.305	0.160	0.176
1008	0.183	0.313	0.428	1.115
1010	0.420	0.359	0.466	4.336
1012	0	0.695	0	4.947
1014	0	0.260	0	3.008
1016	0.137	0.595	0.168	0.519
1018	0.290	0.534	0	0.168
1020	0.260	0.443	0.137	0.076
1022	0	0.741	0.893	0.588
1024	0	7.764	2.596	0.099
1026	0	15.459	0.908	2.970
1028	0	9.489	0	18.772
1030	2.771	4.886	0	66.539
1032	0	12.260	2.603	77.281
1034	0.466	11.085	2.389	36.720
1036	0.893	5.046	3.038	14.368
1038	2.328	0.038	1.137	13.169
1040	6.367	0	0	24.704
1042	8.817	0	0	21.826
1044	13.390	0	0.565	9.024
1046	8.650	2.473	7.535	3.077
1048	2.466	10.444	12.406	2.458
1050	0.954	14.032	5.443	2.191
1052	0	6.894	2.176	0
1054	0	3.542	1.397	0.557
1056	0.786	6.726	0.466	1.756
1058	2.802	12.749	0.412	1.496
1060	36.003	13.665	0	1.321
1062	100	8.504	0	2.191
1064	83.434	1.099	1.458	0.634
1066	23.765	2	1.428	0.260
1068	4.710	4.855	0	6.153
1070	7.962	8.054	2.779	26.101
1072	25.063	2.832	6.306	69.150
1074	37.988	5.390	3.328	40.423
1076	18.276	10.321	1.725	15.818
1078	2.519	28.117	0	9.031
1080	0	54.508	0	5.436
1082	11.993	77.517	0.229	6
1084	46.423	80.357	4.581	11.894
1086	39.743	50.027	7.298	2.619
1088	10.558	27.926	13.467	0
1090	0	33.522	41.683	8.253
1092	1.435	24.284	67.944	0
1094	1.985	5.733	39.721	0
1096	1.107	0.282	13.222	0
1098	0	0	52.065	4.115
1100	1.420	0.115	70.868	0
1102	0.298	0.542	22.299	0
1104	0.176	0.298	2.611	0.580
1106	0	0.069	1.351	3.412
1108	0.008	0.809	1.191	9.054
1110	0.145	1.374	0.878	9.886
1112	0.336	3.107	0	6.298
1114	0.191	4.626	0	3.748
1116	0.695	1.489	0	3.321
1118	0.160	0	0.267	3.382
1120	0.313	0	0.336	0.473
1122	0.397	0	0.145	0
1124	0	0.160	0.267	0
1126	0	0.244	0.145	0.137
1128	0	0.168	0.061	0
1130	0	0.160	0.076	0
1132	0.069	0	0	0.008
1134	0.069	0	0	0
1136	0	0.061	0	0.046
1138	0.046	0.046	0	0.008
1140	0.046	0.023	0	0.069
1142	0	0.122	0	0.046
1144	0	0.076	0	0
1146	0.031	0	0.023	0.076
1148	0.061	0.023	0.008	0.053
1150	0.031	0.053	0	0.122

Biological Application: Glycosylceramides

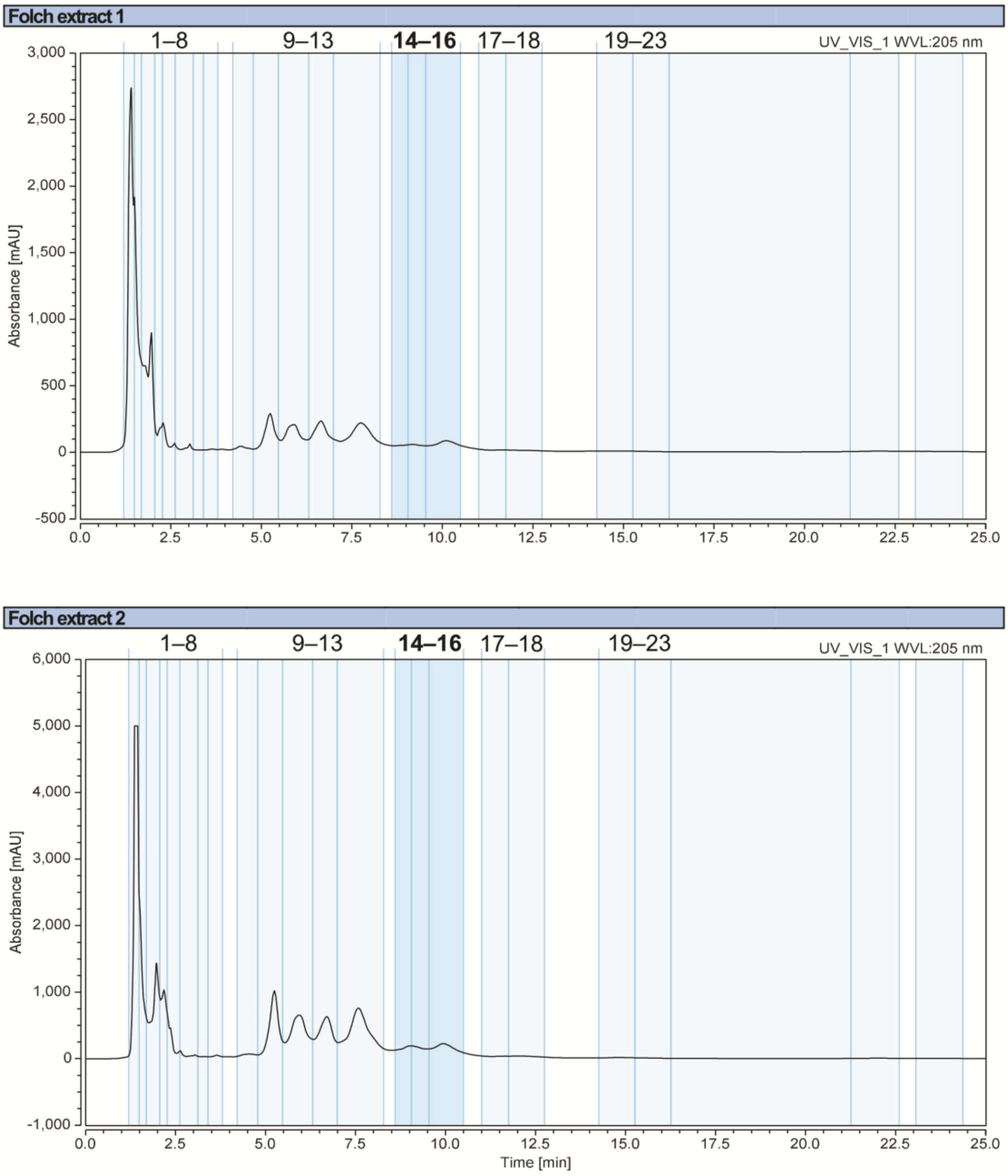

Supplementary Fig. 15 Reversed-phase HPLC chromatograms of Folch extracts **1** and **2** averaged from four injections. Glycosylceramides (809.7 amu) were detected in both samples in the highlighted fractions 14–16 (most abundant in fraction 15).

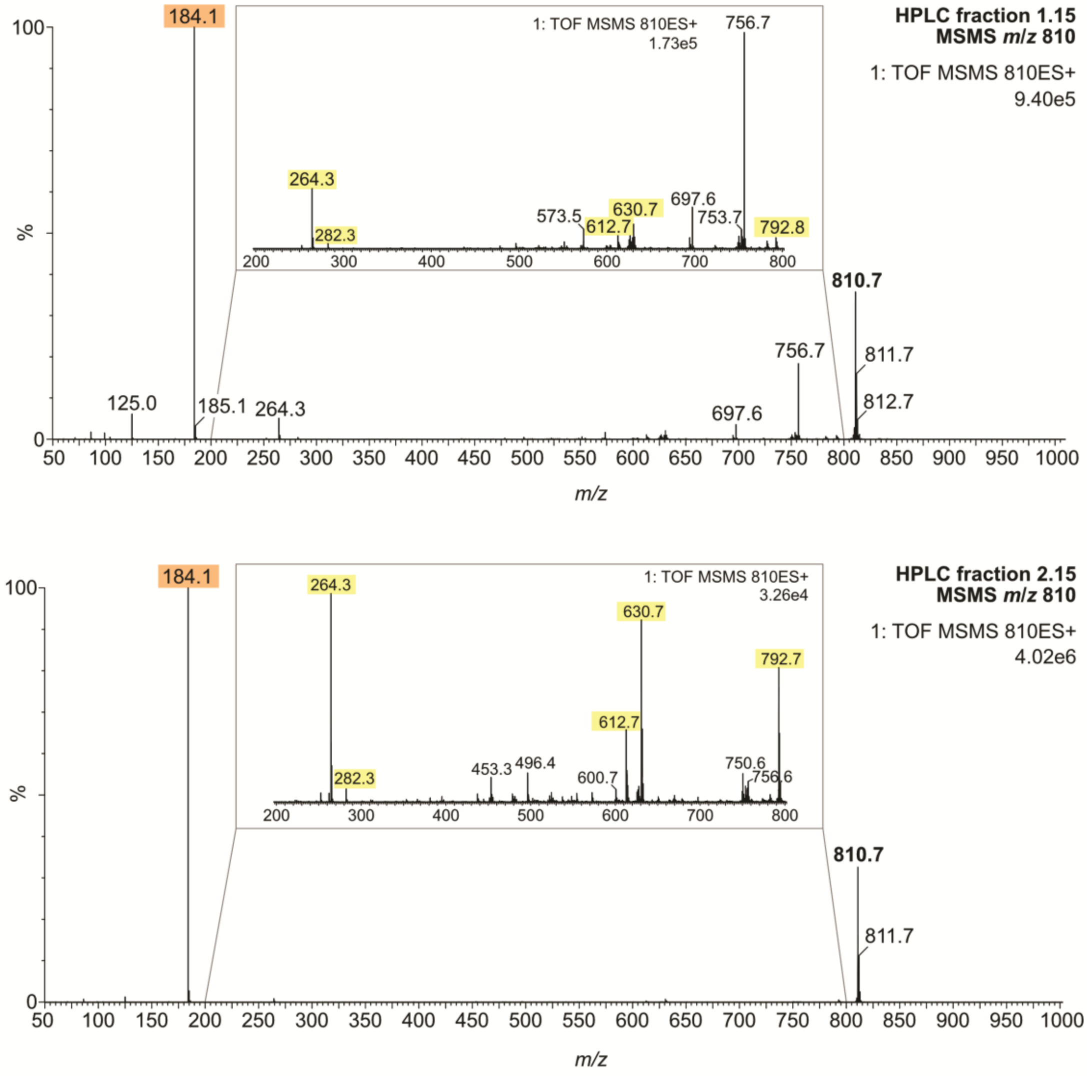

Supplementary Fig. 16 MS/MS spectra of *m/z* 810 precursor ions obtained from the HPLC fraction 15 of Folch extracts **1** (top) and **2** (bottom) by CID (25 V acceleration voltage). The main fragment is phosphocholine (*m/z* 184), originating from protonated phosphatidylcholines. The characteristic fragments of glycosylceramides are highlighted in yellow (*cf.* Supplementary Fig. 1). Source data are provided as a Source Data file.

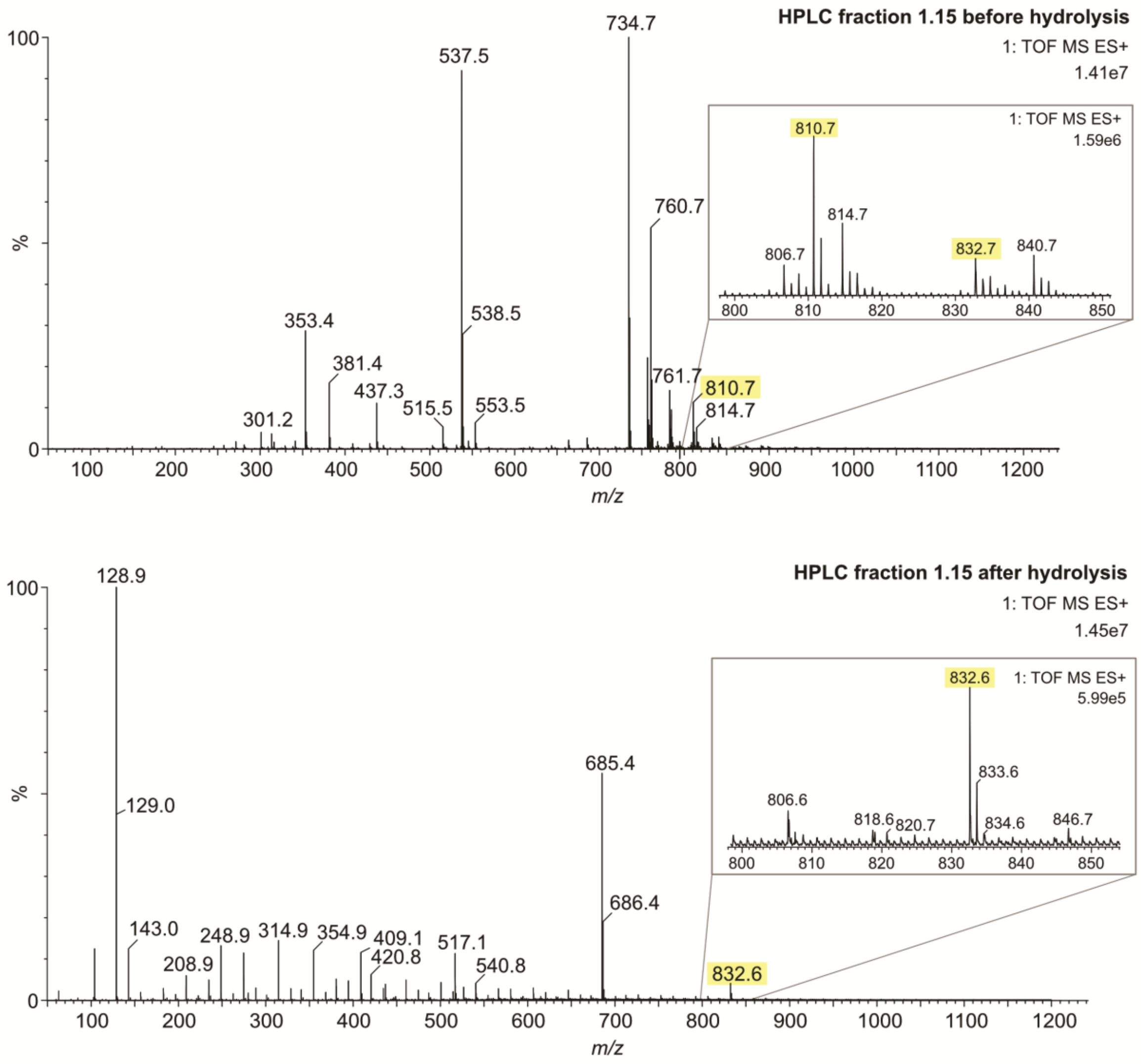

Supplementary Fig. 17 MS spectra of chromatographic fraction 15 from Folch extract **1** before (top) and after hydrolysis (bottom). Before the hydrolysis, the detection of protonated glycosylceramides (*m/z* 810) is hindered by isobaric phosphatidylcholines. After the hydrolytic removal of phosphatidylcholines, the $[M+Na]^+$ adduct of glycosylceramides (*m/z* 832) can be isolated. Source data are provided as a Source Data file.

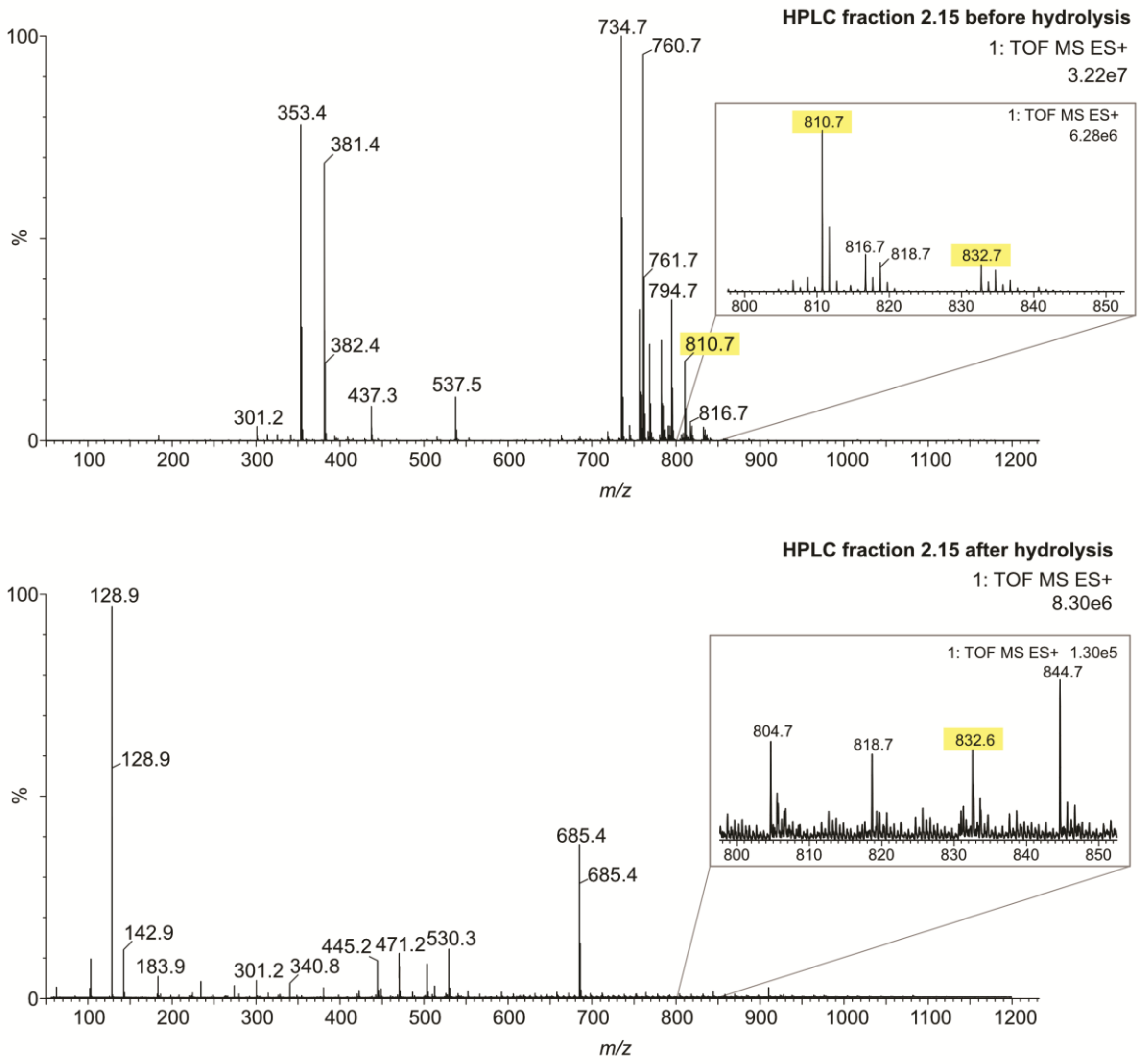

Supplementary Fig. 18 MS spectra of chromatographic fraction 15 from Folch extract **2** before (top) and after hydrolysis (bottom). Before the hydrolysis, the detection of protonated glycosylceramides (*m/z* 810) is hindered by isobaric phosphatidylcholines. After the hydrolytic removal of phosphatidylcholines, the $[M+Na]^+$ adduct of glycosylceramides (*m/z* 832) can be isolated. Source data are provided as a Source Data file.

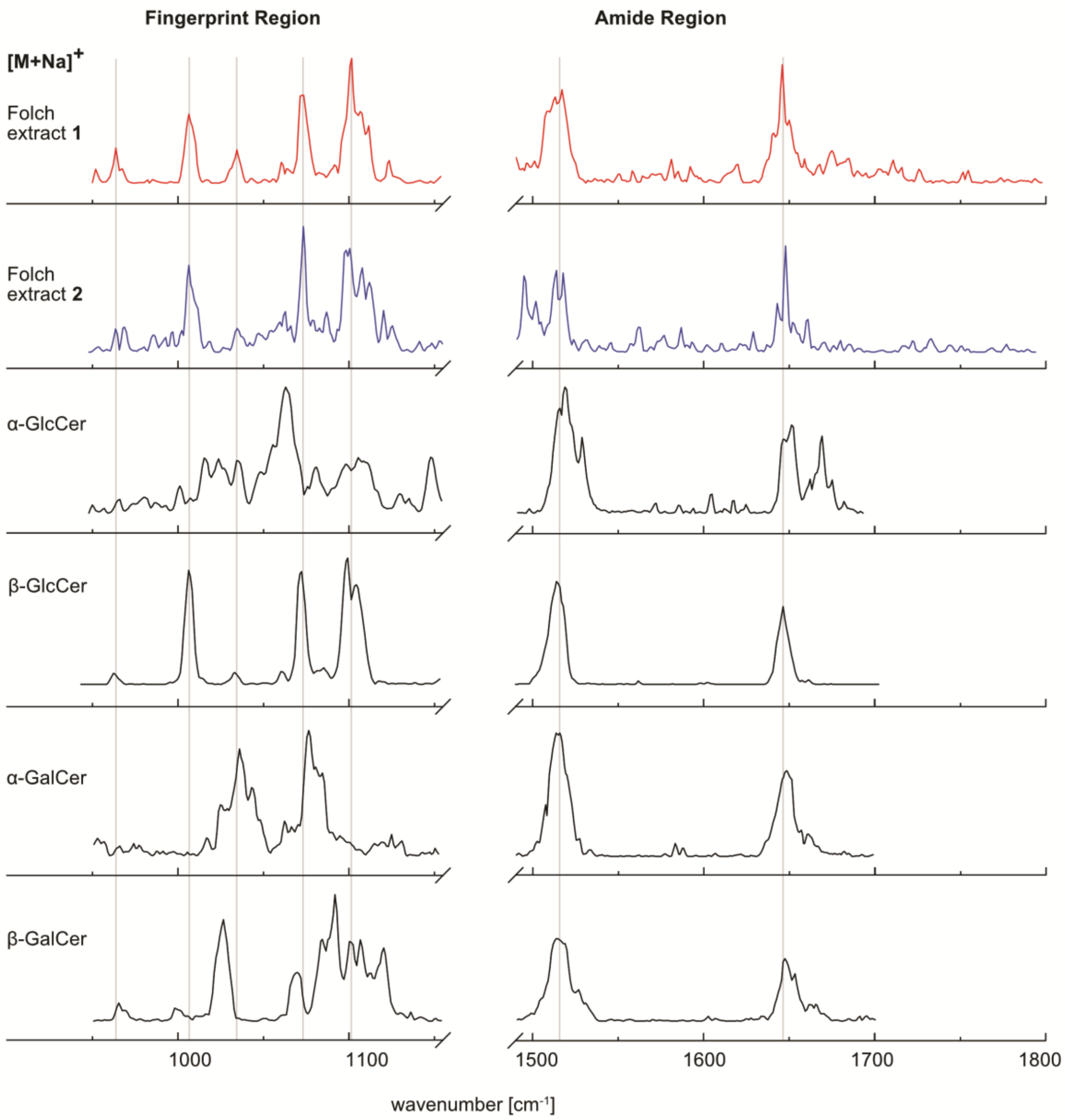

Supplementary Fig. 19 IR spectra of sodiated glycosylceramides (*m/z* 832) from Folch extracts **1** (red) and **2** (blue) after hydrolysis, compared with reference spectra of α- and β-Glc/GalCer. The spectra were recorded in the diagnostic fingerprint region and the amide region. The best spectral match is provided by the reference spectrum of β-GlcCer. Source data are provided as a Source Data file.

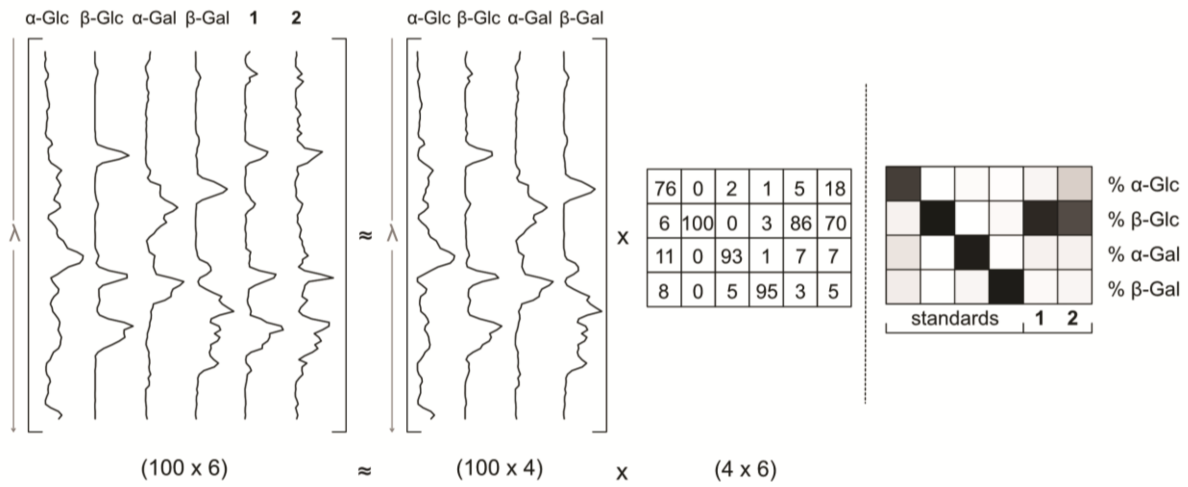

Supplementary Fig. 20* Graphical representation of NMF to deconvolute the IR spectra of of sodiated glycosylceramides (d18:1/24:1) from Folch extracts **1** and **2**. The (100 x 6) input matrix contains experimental spectra of the two biological samples and of the four standards (α- and β-Glc/GalCer) in the fingerprint region (950–1150 cm^{-1}). The output contains the four spectra of α- and β-Glc/GalCer in a (100 x 4) matrix multiplied by their relative contributions to each of the input spectra. The weighting factors were converted into the corresponding ratio of black and white in the adjacent heatmap representation (white = 0 %, black = 100 % glycolipid).

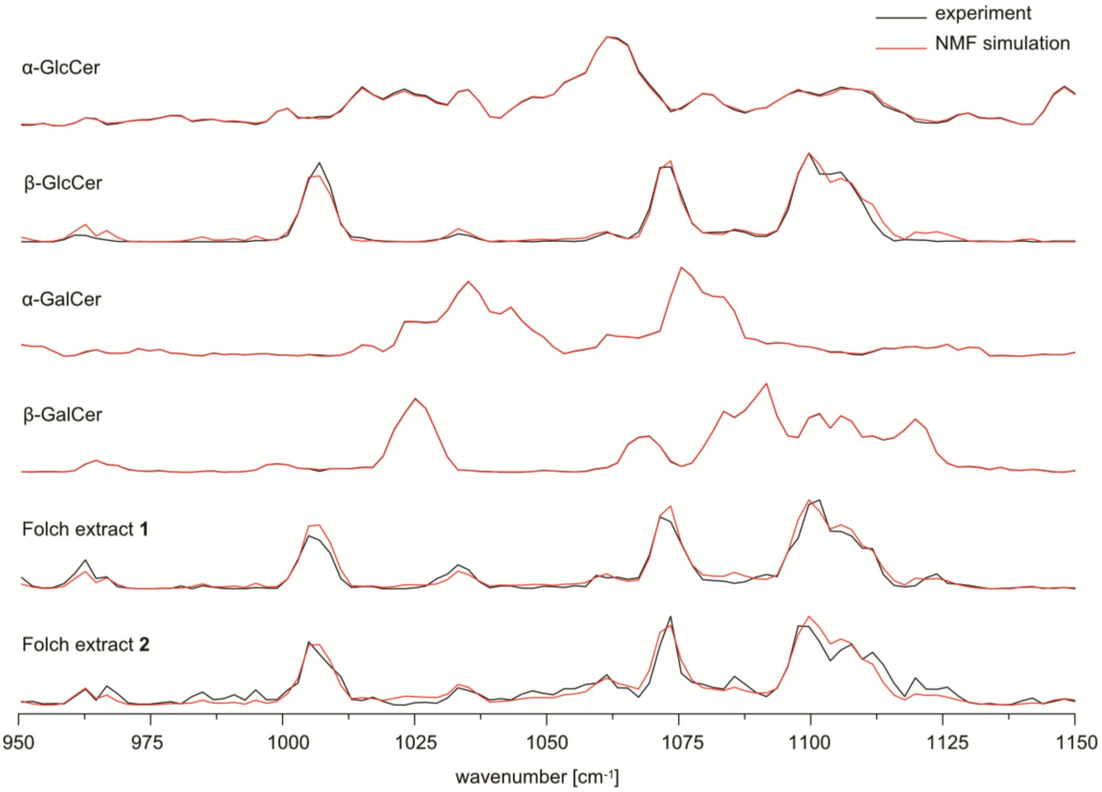

Supplementary Fig. 21 Comparison of NMF-simulated spectra of synthetic and biological glycosylceramides (red) with the experimental IR spectra (black, input spectra from Supplementary Table 6). The spectra were simulated by matrix factorization of the output matrix (Supplementary Table 6) and the weighting factors depicted in Supplementary Fig. 20. Source data are provided as a Source Data file.

* The graphical representation of the input- and output spectra is based on the data in Supplementary Table 6.

Supplementary Table 6 Input- and output matrices of NMF containing normalized spectral intensities. Source data are provided as a Source Data file.

wavenumber	α-GlcCer	β-GlcCer	α-GalCer	β-GalCer	extract 1	extract 2	α-GlcCer	β-GlcCer	α-GalCer	β-GalCer
	Input Matrix (100 x 6)						Output Matrix (100 x 4)			
952	0.871	0.031	12.915	0.530	12.842	3.594	0	5.200	10.393	0.133
954	0.676	0.023	11.156	0.475	3.202	4.357	0	2.141	8.905	0.213
956	2.713	0.023	11.031	0.762	0.784	1.455	0.971	0.213	8.772	0.479
958	0.004	0.069	5.742	0.770	1.372	1.835	0	0.864	4.506	0.479
960	0	2.659	0.172	0.557	5.849	1.415	0	3.564	0.106	0.293
962	3.340	7.786	0.682	1.761	17.156	9.549	1.530	12.209	0.651	1.037
964	8.908	7.166	4.711	9.148	32.514	18.149	4.829	19.697	3.841	6.504
966	8.019	3.981	7.235	13.505	11.797	5.624	4.084	6.690	5.263	9.988
968	1.247	2.209	3.615	8.762	13.332	21.746	0.532	12.621	2.897	6.517
970	2.529	0.493	3.183	7.701	2.418	12.935	1.836	4.987	2.326	5.785
972	5.147	0.451	3.857	3.099	1.000	2.486	3.579	0.811	2.897	2.261
974	6.784	0.466	8.548	1.582	0	1.167	4.404	0	6.645	1.051
976	6.807	0.223	5.973	1.045	0.294	1.012	4.750	0	4.612	0.665
978	9.047	0.027	7.733	1.871	0.400	2.460	6.453	0.027	5.981	1.277
980	11.711	0.238	3.688	0.522	0.654	3.916	9.207	0.638	2.764	0.239
982	11.239	0.253	3.033	0.100	3.072	1.678	8.634	0.825	2.286	0
984	5.024	0	1.074	0.180	0	9.558	4.577	2.793	0.851	0.080
986	6.168	0.014	0.556	1.113	3.497	14.815	5.654	5.732	0.492	0.771
988	7.355	0.090	3.391	1.353	1.830	6.376	5.787	2.155	2.618	0.918
990	4.899	0.107	2.022	0.111	0.621	7.332	4.178	2.288	1.608	0.013
992	4.744	0.014	2.517	0.388	0	11.794	4.324	3.511	2.020	0.239
994	3.913	0.192	1.622	0.887	0	5.707	3.286	1.636	1.236	0.625
996	1.454	1.468	2.520	2.422	1.372	17.274	1.703	6.650	2.033	1.782
998	4.665	1.479	2.908	8.413	0.752	6.186	3.087	2.367	1.927	6.291
1000	16.270	3.010	1.405	9.725	0.642	6.193	12.386	2.101	0.439	7.142
1002	19.580	7.363	1.007	8.475	11.383	17.148	14.967	11.172	0.319	6.012
1004	9.430	31.827	0.566	4.504	33.356	25.057	4.324	32.464	0.266	2.474
1006	8.829	69.563	2.009	3.836	59.744	71.198	2.155	72.683	1.555	1.051
1008	10.247	89.057	1.011	1.306	54.553	57.559	1.104	74.052	0.120	0
1010	10.244	62.756	0.513	3.971	40.239	44.219	3.246	53.810	0	1.370
1012	16.644	19.849	1.291	4.129	11.819	32.218	12.812	21.718	0.651	2.487
1014	31.169	5.585	5.059	4.208	1.638	6.682	24.147	2.500	3.309	2.700
1016	43.605	4.467	12.595	5.764	2.517	5.277	33.181	0.692	9.091	3.724
1018	35.387	1.718	11.312	5.614	2.599	9.109	27.207	1.569	8.307	3.764
1020	29.986	0.755	5.428	18.291	0.143	4.351	22.285	0	3.030	13.486
1022	36.997	0.454	12.912	47.458	0.220	0.474	24.187	0	7.496	35.391
1024	41.408	0.236	39.000	66.295	0.237	0	23.123	0	27.392	49.409
1026	35.558	0.251	39.152	82.792	1.115	2.976	17.681	0	27.033	62.084
1028	33.938	0.903	37.880	71.659	3.297	3.262	17.482	0	26.634	53.705
1030	26.662	2.532	38.837	44.772	11.233	2.313	13.291	1.889	28.894	33.370
1032	23.819	4.634	51.391	17.374	16.444	8.033	11.521	6.796	40.257	12.422
1034	38.395	9.164	70.653	2.577	27.202	19.570	22.018	14.896	56.246	0.745
1036	40.626	7.141	83.932	2.181	21.658	16.478	22.644	10.480	66.759	0.412
1038	27.625	3.381	71.206	1.035	8.498	12.346	14.408	4.229	56.645	0
1040	10.386	0.389	50.154	0.780	1.034	5.926	3.100	0.160	39.978	0.146
1042	8.665	0.201	47.501	0.215	3.130	7.448	2.022	1.556	37.945	0
1044	18.495	0.863	55.164	0.094	3.372	5.870	8.901	0.386	43.872	0
1046	26.897	0.635	40.184	0.042	0.792	12.279	17.934	1.357	31.765	0
1048	32.014	0.250	29.165	0.990	0.602	16.084	23.562	2.367	22.900	0.226
1050	31.517	0.951	21.985	2.204	3.001	13.415	23.495	2.859	17.118	1.157
1052	35.339	1.149	10.440	1.635	2.563	11.770	27.779	2.301	7.815	0.758
1054	48.264	0.908	2.756	0.780	1.461	18.647	39.673	3.391	1.528	0.040
1056	54.097	0.625	5.639	0.457	3.575	18.450	43.944	3.431	3.775	0
1058	59.432	1.618	7.684	1.194	2.611	23.174	48.281	4.602	5.303	0.186
1060	83.982	5.977	10.125	4.021	15.113	24.826	66.654	9.443	6.871	1.875
1062	100	10.210	24.479	5.153	12.498	34.143	78.309	11.664	17.982	2.407
1064	98.785	7.210	22.187	10.792	13.024	19.515	76.406	5.799	15.869	6.770
1066	90.907	3.462	21.690	31.617	10.642	19.150	69.036	3.591	14.753	22.783
1068	61.718	11.207	20.571	39.359	12.574	8.917	43.585	6.105	13.809	28.808
1070	46.587	36.789	24.246	40.616	37.544	24.742	28.378	31.746	17.012	29.233
1072	32.792	83.770	28.339	29.324	80.870	67.293	14.063	81.673	21.172	19.564
1074	15.862	84.250	66.813	11.881	75.573	100	0	91.289	53.149	6.278
1076	20.198	52.869	100	6.627	50.452	35.979	0	46.815	79.491	2.780
1078	27.684	20.778	89.920	10.496	26.775	25.758	10.125	21.599	71.304	6.517
1080	36.171	10.344	71.806	27.080	10.216	23.497	19.424	10.334	55.861	19.471
1082	34.662	10.560	67.394	46.541	9.434	20.002	17.069	8.911	51.475	34.301
1084	23.865	11.075	66.634	68.672	8.718	18.051	6.719	8.698	50.066	51.245
1086	17.934	13.029	50.535	61.551	6.439	32.404	5.162	14.537	37.626	45.978
1088	14.432	10.554	20.648	69.822	8.123	23.691	4.563	12.289	13.437	52.535
1090	19.004	6.228	18.956	84.432	12.972	14.389	6.852	8.631	11.483	63.654
1092	20.759	6.293	14.123	100	16.229	9.715	7.171	8.033	6.951	75.477
1094	28.177	12.529	15.138	62.698	13.835	19.653	16.005	13.020	9.131	46.922
1096	35.133	35.261	14.297	41.040	41.633	42.917	21.367	39.859	9.503	29.765
1098	39.277	76.518	11.557	39.018	56.936	89.109	23.881	77.337	7.124	27.278
1100	37.534	100	10.876	61.114	94.756	88.452	16.790	100	5.874	43.385
1102	34.677	76.468	8.073	65.669	100	71.273	15.047	86.833	3.934	47.348
1104	39.076	76.226	4.885	49.695	64.990	49.807	20.568	66.990	1.143	35.365
1106	43.384	78.492	4.815	63.572	64.437	62.320	23.735	71.353	0.478	45.832
1108	41.355	63.856	2.360	57.299	59.279	68.436	24.666	65.926	0	41.390
1110	40.440	44.800	1.491	39.591	45.746	51.461	26.555	48.052	0	28.422
1112	35.531	22.861	4.958	40.570	45.230	59.348	24.759	41.907	2.512	29.712
1114	22.618	8.257	8.905	36.314	16.149	45.316	16.125	21.772	5.715	27.065
1116	17.882	1.341	8.449	38.815	3.790	24.553	12.040	7.900	5.064	29.220

1118	13.762	2.389	10.222	47.699	2.127	9.751	6.865	2.886	5.994	35.990
1120	5.003	2.356	10.832	59.902	3.717	30.878	0.213	10.972	6.326	45.366
1122	3.128	1.977	9.128	49.054	12.313	18.338	0	9.855	5.276	36.947
1124	3.197	0.617	10.033	21.494	16.786	18.023	0.173	11.424	7.523	16.146
1126	5.297	0.851	13.360	10.143	6.350	20.299	3.033	8.459	10.446	7.528
1128	12.239	0.749	6.962	6.004	5.346	10.805	9.047	4.602	5.290	4.349
1130	14.561	0.800	9.986	6.666	3.905	3.338	10.098	1.330	7.549	4.801
1132	9.326	0.992	9.723	5.489	1.698	1.222	5.947	0.319	7.403	3.977
1134	8.369	0.808	0.461	3.267	0.138	1.976	6.519	0.346	0.093	2.367
1136	9.116	0.430	0.687	5.726	0.336	0.469	6.759	0	0.133	4.216
1138	5.086	0.612	0.354	2.534	0.955	0.871	3.832	0.452	0.106	1.849
1140	1.486	1.235	0	2.179	1.998	4.837	1.144	2.687	0	1.596
1142	1.796	0.192	1.155	3.237	2.069	7.905	1.503	3.245	0.864	2.421
1144	15.109	0.105	2.073	2.225	0.751	1.540	11.761	0	1.316	1.490
1146	35.619	0.809	1.984	0.452	0.513	4.403	28.484	0	0.944	0
1148	44.480	0.850	1.925	0	1.347	6.965	35.855	0	0.797	0
1150	36.216	0.689	4.088	1.930	0.319	4.280	28.591	0	2.539	0.971

Computed IR Spectra and Structures

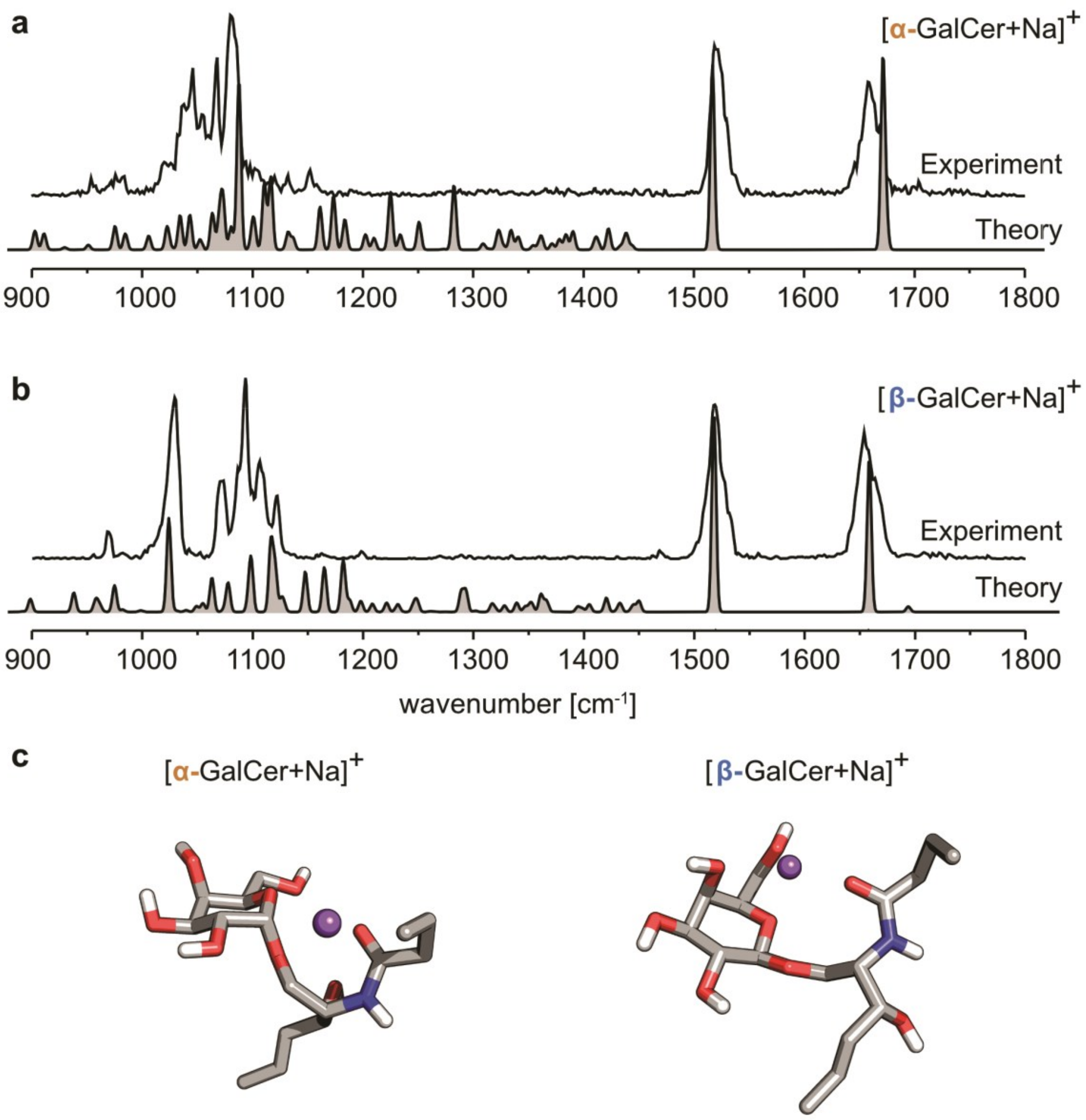

Supplementary Fig. 22 Experimental and computed harmonic IR spectra of the lowest-energy conformers of $[M+Na]^+$ ions with truncated lipid chains of α-GalCer (**a**) and β-GalCer (d18:1/24:1(15*Z*)) (**b**). The corresponding three-dimensional structures with trimmed lipid chains are depicted in panel **c**.

Cartesian Coordinates of Reoptimized Conformers

alpha GalCer $[M+Na]^{+}$

C	-3.581851	-0.751434	0.155061
C	-2.387941	-0.707624	1.100383
O	-1.296734	-1.492680	0.608646
C	-0.793590	-1.075425	-0.631097
C	-1.903998	-1.086021	-1.673578
C	-3.112079	-0.275208	-1.217119
H	-4.354000	-0.062164	0.520765
H	-2.073145	0.345070	1.171443
H	-0.005913	-1.788572	-0.899581
H	-2.214569	-2.136251	-1.780345
H	-2.818080	0.777486	-1.149625
O	-1.377957	-0.586693	-2.876226
H	-2.112062	-0.485686	-3.491399
O	-4.133021	-0.343204	-2.180220
H	-4.550449	-1.208506	-2.090408
O	-4.104107	-2.057503	-0.009321
H	-4.871434	-2.181721	0.551308
C	-2.655925	-1.211483	2.494625
H	-2.838915	-2.290778	2.465563
H	-3.532821	-0.697722	2.906397
O	-1.493103	-0.914900	3.271113
H	-1.614723	-1.246527	4.163551
O	-0.219882	0.194778	-0.409044
C	0.768713	0.612943	-1.339015
H	1.189152	-0.248157	-1.867262
H	0.286995	1.246292	-2.087261
C	1.904829	1.405689	-0.682550
H	2.240037	2.083816	-1.474455
C	1.499373	2.357467	0.456778
H	2.362106	3.016266	0.624517
N	3.095633	0.622233	-0.360824
H	3.972687	1.003717	-0.677288
C	3.156920	-0.577434	0.249880
O	2.158579	-1.164902	0.666572
C	4.527651	-1.195369	0.377916
H	5.296145	-0.557739	-0.072527
H	4.744432	-1.258930	1.449778
C	4.561809	-2.592743	-0.243908
H	3.803518	-3.208882	0.248540
H	5.532582	-3.044417	-0.018839
C	4.330227	-2.576955	-1.748056
H	5.080290	-1.965954	-2.261184
H	3.342197	-2.175405	-1.998670
H	4.385865	-3.585237	-2.164721
O	1.275062	1.613730	1.668074
H	1.152993	2.266189	2.363680
C	0.308080	3.190384	0.105187
H	-0.623189	2.649979	-0.053852
C	0.352872	4.517569	-0.007628
H	1.305429	5.021964	0.164059
C	-0.799576	5.390726	-0.363103
H	-0.590193	5.951574	-1.280203
H	-1.715698	4.815242	-0.513477
H	-0.979275	6.134038	0.420767
Na	0.455165	-0.551283	1.930643

beta GalCer $[M+Na]^+$

C	-3.81481000	-0.63343200	0.02094100
C	-2.91707300	-0.78500200	1.24033100
O	-1.59959200	-0.38961400	0.89001600
C	-1.48343700	0.98726900	0.53639900
C	-2.45375300	1.36591700	-0.57928900
C	-3.86112800	0.84053700	-0.35344600
H	-4.82801100	-0.98056300	0.25753600
H	-3.31651600	-0.11943600	2.02127400
H	-1.65229000	1.59781700	1.43896000
H	-2.06551000	0.90075000	-1.49555600
H	-4.33443200	1.40632000	0.46199900
O	-2.54862300	2.76000300	-0.73000200
H	-1.69960500	3.09597400	-1.03067700
O	-4.62186400	0.93045300	-1.53406100
H	-4.59001200	1.83842400	-1.85146000
O	-3.27747900	-1.38549900	-1.05984300
H	-3.76665300	-1.11117200	-1.84567400
C	-2.83841300	-2.16348900	1.86305600
H	-3.85836400	-2.51378000	2.05873500
H	-2.30467600	-2.08139300	2.81660700
O	-2.15156400	-3.05278000	0.99392500
H	-2.15739100	-3.93290100	1.37477300
O	-0.22778300	1.17286600	0.00352700
C	0.87463500	0.93125100	0.87793600
H	1.04423400	1.80670400	1.51241100
H	0.66332900	0.06436000	1.50592500
C	2.08462200	0.64024600	-0.01657900
H	1.69746800	0.50123900	-1.02978600
C	3.10753100	1.77722600	-0.03787400
H	3.88215500	1.49575200	-0.76850900
N	2.77113500	-0.57930000	0.36093000
H	3.60843400	-0.48296500	0.91706600
C	2.28898200	-1.79663500	0.07747900
O	1.21841600	-1.94120000	-0.52604900
C	3.11333600	-2.97980900	0.50996500
H	4.01452200	-2.65006900	1.03695500
H	2.50680300	-3.54876200	1.22280000
C	3.48690600	-3.86572900	-0.67987300
H	2.56686400	-4.20305300	-1.16715300
H	3.98567500	-4.76046300	-0.29570000
C	4.38358200	-3.16001000	-1.68575100
H	5.31384400	-2.82020300	-1.21917700
H	3.88858800	-2.28605500	-2.12176200
H	4.65197600	-3.82749400	-2.50771000
O	3.66511800	1.80540600	1.26267900
H	4.34087700	2.48762500	1.29720400
C	2.48957500	3.07153000	-0.47205400
H	2.20572600	3.11805900	-1.52288700
C	2.28888500	4.12410500	0.31681200
H	2.58113000	4.05391200	1.36428600
C	1.68760700	5.41588100	-0.12064900
H	2.38340700	6.24536700	0.04291900
H	1.41930300	5.40102600	-1.17958500
H	0.78971900	5.64579700	0.46323400
Na	-0.96762300	-2.00926800	-0.69723000

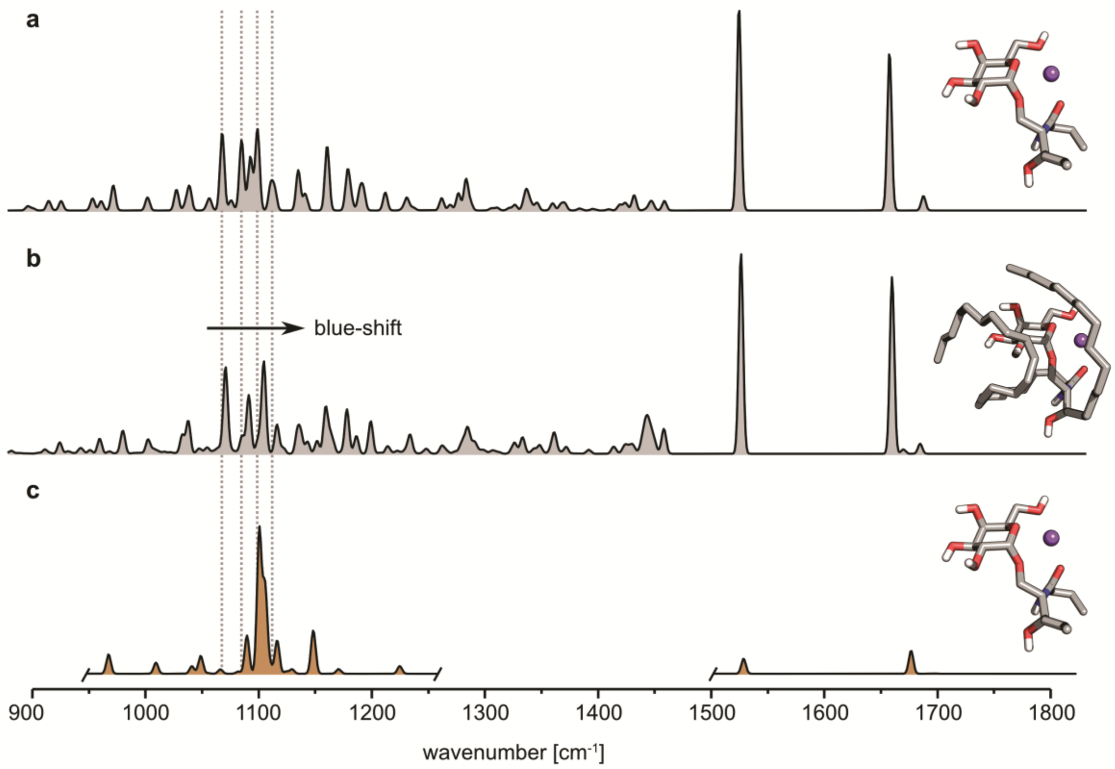

Supplementary Fig. 23 Comparison of the theoretical spectrum of a low-energy conformer of [α-GalCer+Na]$^+$ (**a**) with lipid chains truncated to include only relevant functional groups of the lipid chain and calculated within harmonic approximation; (**b**) with full lipid chains attached to the same core-structure of the ion and calculated with harmonic approximation and (**c**) with lipid chains truncated and calculated including anharmonic effects. Attachment of the full lipid chains results in only minor changes to the diagnostic region (average blue-shift of 5 cm^{-1}) of the IR spectrum between 1000 and 1150 cm^{-1}. Anharmonicity of the potential has a larger impact on the IR spectrum.

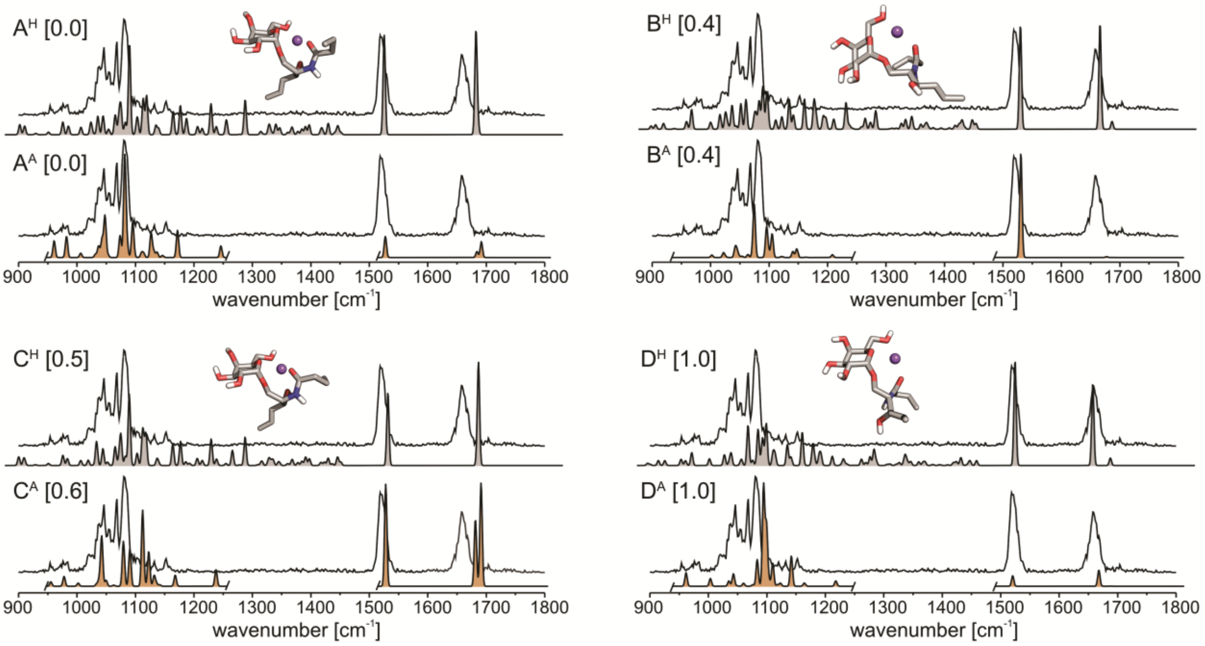

Supplementary Fig. 24 Comparison of the harmonic (gray, scaled by 0.965) and anharmonic (orange, red-shifted by 20 cm^{-1}) theoretical IR spectra of four low-energy conformers of [α-GalCer+Na]$^+$ with lipid chains truncated to include only relevant functional groups. The experimental spectrum of the [α-GalCer+Na]$^+$ cation is shown as black trace. The numbers next to the label indicate the relative electronic energy (in kcal mol^{-1}), including harmonic (^{H}) or anharmonic (^{A}) zero-point energy corrections. Evaluation of anharmonic vibrations only considered bands in the 1000–1150 cm^{-1} diagnostic region, amide I and II, and C=C vibrations.

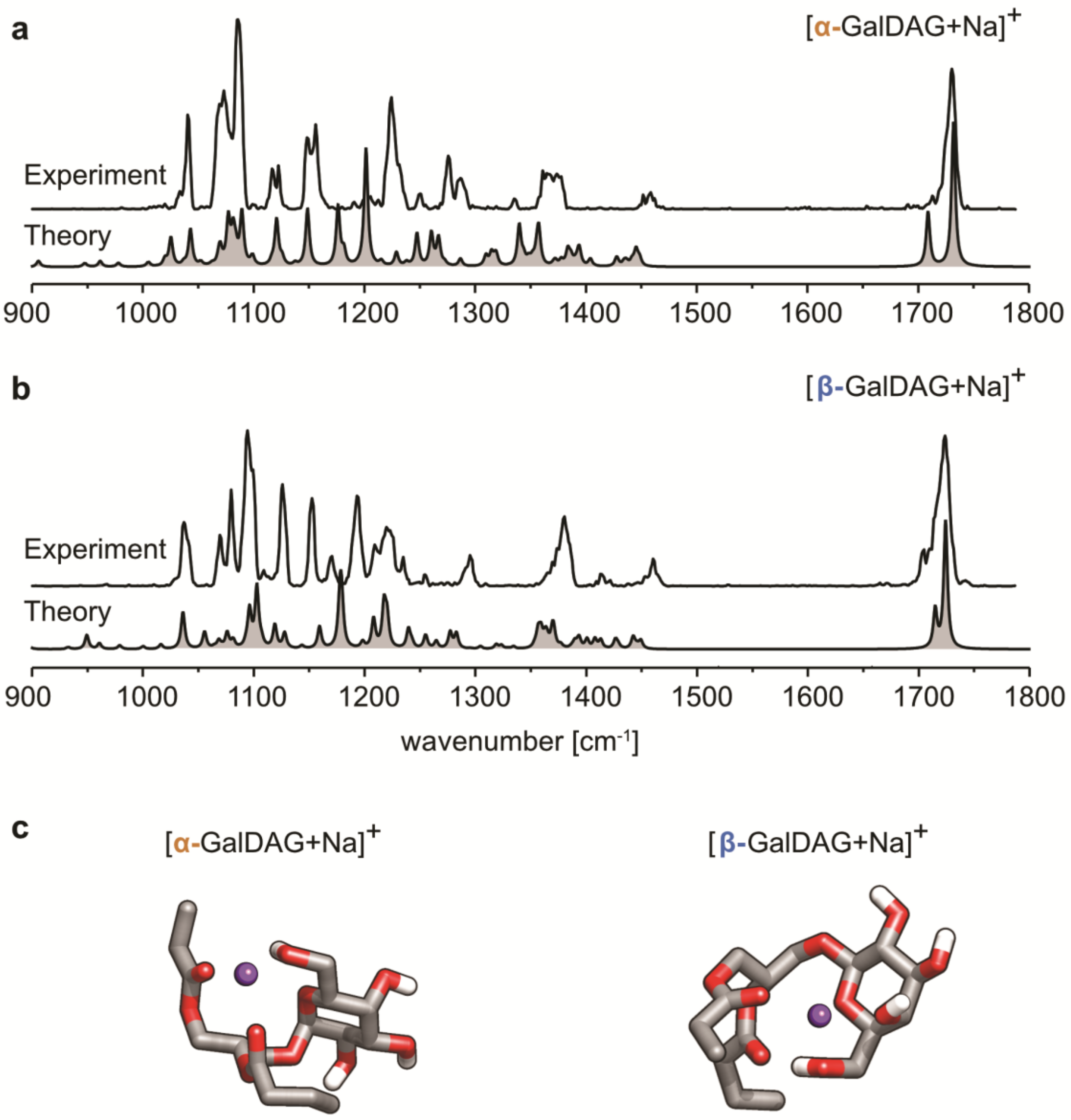

Supplementary Fig. 25: Experimental and computed harmonic IR spectra of $[M+Na]^+$ ions of α-Gal DAG (**a**) and β-Gal DAG (14:0/14:0) (**b**). The anomers are distinguishable by characteristic C–C, C–O and C–H vibrations, which are difficult to model. The C=O frequencies, however, are in good agreement with the experiment and consist of in- and out-of-phase vibrations of the two ester groups. The corresponding three-dimensional structures with trimmed lipid chains are depicted in panel **c**.

Cartesian Coordinates of Reoptimized Conformers

alpha GalDAG $[M+Na]^+$

C	-2.74570	-0.98040	1.27550
C	-1.24810	-0.74730	1.14980
C	-1.21420	-1.46390	-1.13620
C	-2.70560	-1.76310	-1.09360
C	-3.40290	-0.85400	-0.09240
H	-1.08830	0.31170	0.90360
H	-2.82630	-2.79890	-0.76670
H	-3.32190	0.18830	-0.43430
O	-0.63530	-1.55110	0.13910
O	-1.07310	-0.19030	-1.71760
O	-3.29610	-1.66880	-2.36610
H	-3.03050	-0.82420	-2.74660
O	-4.75170	-1.22100	0.06830
H	-5.11740	-1.40470	-0.80360
C	-0.51600	-1.09030	2.43120
H	-0.80080	-0.39640	3.22420
H	-0.77520	-2.10340	2.74500
O	0.89630	-0.97650	2.25340
H	1.13850	-1.65010	1.60990
C	0.20610	0.11570	-2.22770
H	0.06110	0.74890	-3.10750
H	0.72680	-0.79790	-2.54030
C	1.06570	0.84930	-1.21930
H	1.01080	0.34670	-0.25370
C	2.51690	0.97690	-1.66310
H	3.05730	1.64640	-0.99150
H	2.58300	1.35640	-2.68360
O	0.52880	2.17610	-1.09320
O	3.15490	-0.30290	-1.69330
C	0.60860	2.78720	0.08730
C	3.60450	-0.79800	-0.54060
O	3.44300	-0.23060	0.52250
O	1.15620	2.28630	1.05300
C	-0.08340	4.11030	0.10630
H	0.54710	4.80010	0.67230
H	-0.19260	4.48080	-0.91490
C	-1.45930	3.98930	0.78230
H	-1.85330	5.00180	0.90600
H	-1.32300	3.58640	1.79160
C	-2.44760	3.14310	-0.00550
H	-2.08120	2.12420	-0.16460
H	-3.40510	3.07840	0.51670
H	-2.63550	3.57390	-0.99330
C	4.32240	-2.09630	-0.72640
H	3.72890	-2.71100	-1.41060
H	5.24150	-1.86230	-1.27810
C	4.62910	-2.81360	0.57480
H	5.23590	-2.19300	1.23650
H	3.71460	-3.08450	1.10890
H	5.18030	-3.73340	0.37320
Na	2.32370	0.76880	2.18240
H	-0.68750	-2.20880	-1.74320
O	-2.96950	-2.25520	1.82080
H	-3.90500	-2.45160	1.69150
H	-3.15420	-0.19850	1.93530

beta GalDAG $[M+Na]^+$

C	-3.16620	-0.52630	-1.46810
H	-3.67150	-1.00550	-2.31550
C	-4.05100	-0.64080	-0.23210
C	-3.32010	-0.19360	1.02820
H	-3.13560	0.88870	0.96410
O	-4.16310	-0.49960	2.10740
H	-3.79200	-0.12940	2.91300
C	-1.96420	-0.87340	1.13230
O	-1.27530	-0.29990	2.18440
C	0.00540	-0.86570	2.43470
C	1.07200	-0.10230	1.65620
C	1.44210	1.20640	2.31320
H	1.97200	1.04120	3.25180
H	0.53840	1.79390	2.49170
O	2.36090	1.94810	1.49980
C	1.88100	2.54830	0.41840
O	0.70070	2.51490	0.11970
Na	-0.91020	1.68770	-1.14350
O	0.00900	-0.04000	-2.29810
H	0.83280	-0.26330	-1.83570
C	-0.80250	-1.19770	-2.33390
C	-1.84400	-1.22820	-1.20650
H	-2.08600	-2.28350	-1.00720
O	-1.23140	-0.65120	-0.06250
H	-0.17370	-2.08880	-2.25370
H	-1.31300	-1.23400	-3.29990
O	-5.17480	0.16900	-0.47770
H	-5.72880	0.17640	0.30930
C	2.94500	3.25800	-0.35730
C	2.48250	3.73650	-1.72050
H	2.18380	2.89740	-2.35480
H	1.63770	4.42310	-1.63490
H	3.29250	4.26330	-2.22790
H	3.28260	4.09430	0.26710
H	3.80400	2.58390	-0.42800
H	0.69060	0.07930	0.65800
O	2.23770	-0.92590	1.54400
C	2.74280	-1.10810	0.31380
O	2.25050	-0.62930	-0.68600
C	3.98760	-1.94000	0.33880
C	4.20390	-2.72760	-0.94710
C	3.16060	-3.81590	-1.15500
H	3.16450	-4.53280	-0.32790
H	3.35090	-4.37210	-2.07560
H	2.15310	-3.39290	-1.22960
H	4.20200	-2.03630	-1.79480
H	5.20170	-3.17400	-0.90790
H	4.81290	-1.23530	0.50540
H	3.95850	-2.58840	1.21860
H	0.17060	-0.81210	3.51380
H	0.02990	-1.91890	2.13470
H	-2.07500	-1.96120	1.28370
H	-4.34720	-1.69230	-0.10410
O	-2.93760	0.84720	-1.76530
H	-3.80640	1.26980	-1.75110
